# APAF1‐Binding Long Noncoding RNA Promotes Tumor Growth and Multidrug Resistance in Gastric Cancer by Blocking Apoptosome Assembly

**DOI:** 10.1002/advs.202201889

**Published:** 2022-08-17

**Authors:** Qiang Wang, Chen Chen, Xiao Xu, Chuanjun Shu, Changchang Cao, Zhangding Wang, Yao Fu, Lei Xu, Kaiyue Xu, Jiawen Xu, Anliang Xia, Bo Wang, Guifang Xu, Xiaoping Zou, Ruibao Su, Wei Kang, Yuanchao Xue, Ran Mo, Beicheng Sun, Shouyu Wang

**Affiliations:** ^1^ Department of Hepatobiliary Surgery The Affiliated Drum Tower Hospital of Nanjing University Medical School Nanjing 210000 China; ^2^ Department of Hepatobiliary Surgery The First Affiliated Hospital of Anhui Medical University Hefei 230022 China; ^3^ Jiangsu Key Laboratory of Molecular Medicine Medical School of Nanjing University Nanjing 210000 China; ^4^ State Key Laboratory of Natural Medicines Jiangsu Key Laboratory of Drug Discovery for Metabolic Diseases Center of Advanced Pharmaceuticals and Biomaterials School of Life Science and Technology China Pharmaceutical University Nanjing 210000 China; ^5^ Department of Bioinformatics School of Biomedical Engineering and Informatics Nanjing Medical University Nanjing 210000 China; ^6^ Key Laboratory of RNA Biology Institute of Biophysics Chinese Academy of Sciences Beijing 100101 China; ^7^ Department of Gastroenterology The Affiliated Drum Tower Hospital of Nanjing University Medical School Nanjing 210000 China; ^8^ Department of Pathology The Affiliated Drum Tower Hospital of Nanjing University Medical School Nanjing 210000 China; ^9^ Department of Anatomical and Cellular Pathology Institute of Digestive Disease State Key Laboratory of Digestive Disease State Key Laboratory of Translational Oncology Prince of Wales Hospital The Chinese University of Hong Kong Hong Kong SAR 999077 China; ^10^ Center for Public Health Research Medical School of Nanjing University Nanjing 210000 China

**Keywords:** ABL, apoptotic protease‐activating factor 1 (APAF1), apoptosis, drug resistance, gastric cancer, IGF2BP1, m^6^A

## Abstract

Chemotherapeutics remain the first choice for advanced gastric cancers (GCs). However, drug resistance and unavoidable severe toxicity lead to chemotherapy failure and poor prognosis. Long noncoding RNAs (lncRNAs) play critical roles in tumor progression in many cancers, including GC. Here, through RNA screening, an apoptotic protease‐activating factor 1 (APAF1)‐binding lncRNA (ABL) that is significantly elevated in cancerous GC tissues and an independent prognostic factor for GC patients is identified. Moreover, ABL overexpression inhibits GC cell apoptosis and promotes GC cell survival and multidrug resistance in GC xenograft and organoid models. Mechanistically, ABL directly binds to the RNA‐binding protein IGF2BP1 via its KH1/2 domain, and then IGF2BP1 further recognizes the METTL3‐mediated m6A modification on ABL, which maintains ABL stability. In addition, ABL can bind to the WD1/WD2 domain of APAF1, which competitively prevent cytochrome c from interacting with APAF1, blocking apoptosome assembly and caspase‐9/3 activation; these events lead to resistance to cell death in GC cells. Intriguingly, targeting ABL using encapsulated liposomal siRNA can significantly enhance the sensitivity of GC cells to chemotherapy. Collectively, the results suggest that ABL can be a potential prognostic biomarker and therapeutic target in GC.

## Graphical abstract

Schematic illustration shows that lncRNA apoptotic protease‐activating factor 1 (APAF1)‐binding lncRNA (ABL) binds to IGF2BP1, and then IGF2BP1 recognizes the METTL3‐mediated m6A modification on ABL. In addition, ABL interacts with APAF1 to block apoptosome assembly and caspase‐9/3 activation, leading to resistance to cell death in gastric cancer cells. Meanwhile, targeting ABL using encapsulated liposomal siRNA can significantly enhance the sensitivity of gastric cancer cells to chemotherapy.

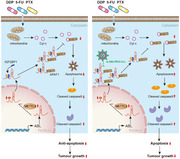

## 1 Introduction

Gastric cancer (GC) is the fifth most common malignancy and the third leading cause of cancer‐related death globally, with ≈40% of new cases and deaths occurring in China each year.^[^ ^1^ ^]^ Chemotherapeutics remain the cornerstone of therapy for advanced GC.^[^ ^1^, ^2^ ^]^ However, drug resistance and unavoidable severe toxicity lead to chemotherapy failure.^[^ ^3^ ^]^ Therefore, there is an urgent need to elucidate more detailed molecular mechanisms and identify effective therapeutic targets for GC treatment.

Cytotoxic drugs, including cisplatin (DDP), 5‐fluorouracil (5‐Fu), and paclitaxel (PTX), are widely used for GC chemotherapy and mainly induce cancer cell apoptosis via the intrinsic apoptosis pathway.^[^ ^1^, ^4^ ^]^ The cytotoxic insults caused by these drugs can activate BH3‐only proteins, which further activate and oligomerize Bax and Bak downstream;^[^ ^5^ ^]^ then oligomerized Bax/Bak promotes the release of cytochrome c (Cyt c) from the mitochondria into the cytosol. Subsequently, apoptotic protease‐activating factor 1 (APAF1) and Cyt c assemble into an oligomeric apoptosome complex, which is responsible for the activation of caspase‐9, and caspase‐9 then activates effector caspases, such as caspase‐3 and caspase‐7, which cleave various cellular proteins, leading to cell death.^[^ ^6^ ^]^ It has been reported that both intracellular nucleotides and transfer RNA (tRNA) can bind to Cyt c to block APAF1 oligomerization, apoptosome formation, and caspase activation,^[^ ^7^ ^]^ which pushed us to consider whether other types of RNAs that can modulate cell apoptosis by binding key components in the intrinsic apoptosis pathway.

Long noncoding RNA (lncRNA) is defined as a class of transcripts longer than 200 nucleotides (nt) without a protein‐coding capacity, and lncRNAs have been identified to modulate various biological processes, including proliferation, metastasis, metabolism, and drug resistance in cancer.^[^ ^8^ ^]^ LncRNAs exert their regulatory functions through specific interactions with proteins via canonical or noncanonical RNA‐binding domains (RBDs),^[^ ^9^ ^]^ which may regulate the expression/activity of partner proteins, spatial conformation, and protein–protein interactions. Our previous studies identified a few novel lncRNAs involved in the progression of cancer.^[^ ^10^ ^]^ Furthermore, targeting lncRNAs by siRNA‐based strategies has demonstrated clinical potential.^[^ ^11^ ^]^ Therefore, it is of great interest to uncover new functions of lncRNAs by dissecting lncRNA–protein interactions in certain biological processes and further explore the potential clinical value of lncRNAs.

In the present study, we identified an APAF1‐binding lncRNA (ABL, also known as *RP3‐512B11.3*) and revealed its biological function in GC. We found IGF2BP1 directly bound and recognized the METTL3‐mediated m^6^A modification on ABL and maintained ABL stability, which caused upregulation of ABL in GC tissues. Then abundant ABL could directly interact with APAF1, a new identified RNA‐binding protein (RBP), to inhibit the intrinsic apoptosis pathway by competitively blocking the interaction of APAF1 with Cyt c, thus blocking apoptosome assembly and caspase‐9/3 activation, which led to resistance to cell death in GC cells. These findings demonstrated that ABL might be a promising predictive biomarker and therapeutic target in GC patients in the clinic.

## 2 Results

### 2.1 ABL Is Upregulated in GC and Predicts a Poor Prognosis in Patients with GC

**Figure 1:**
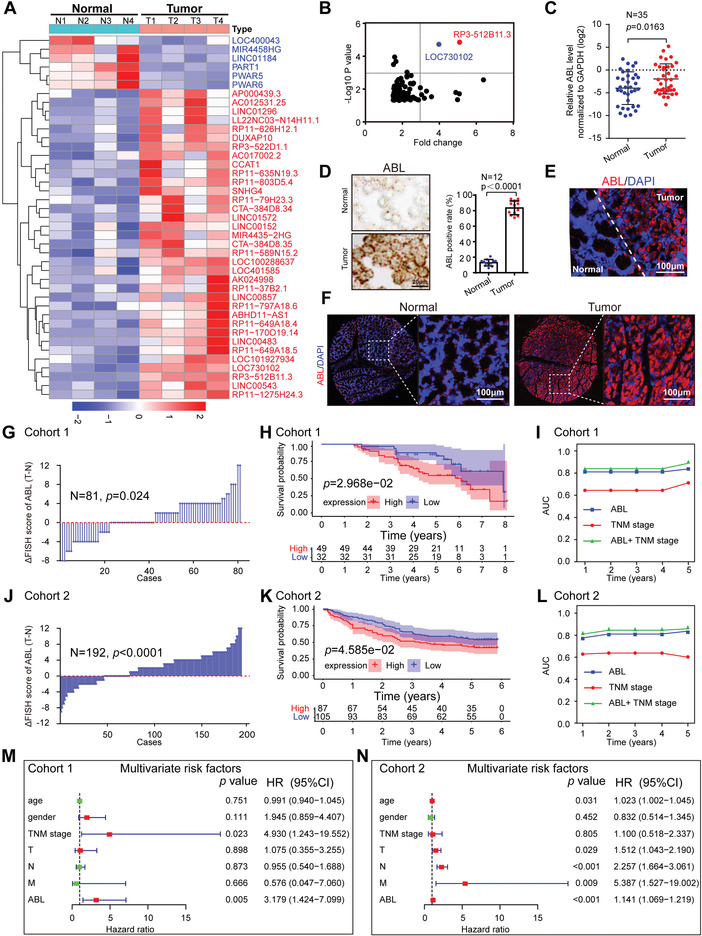
Elevated ABL expression correlates with a poor prognosis in GC patients. A) Hierarchical clustering showing differentially expressed lncRNAs in cancerous GC tissues and matched normal tissues (fold change > 2 or < 0.5, *p* < 0.05; *n* = 4). B) Scatter diagram showing the overlapped upregulated lncRNAs from the significantly differentially expressed candidates ranked by *p*‐value < 0.05 and fold change > 1.5. C) The levels of ABL expression in paired GC and normal gastric mucosal tissues were measured by qRT‐PCR (*n* = 35). D) RNAscope detection of ABL expression in GC and adjacent normal tissues (scale bars = 20 µm, *n* = 12, left panel). Statistical analysis of ABL expression (right panel). E) Representative images of RNA‐FISH staining of GC tissues with an ABL probe. Normal (N) or tumor (T) tissues are marked with dotted lines (scale bars = 100 µm). F) Representative RNA‐FISH images of a tissue microarray (TMA) for cohort 1 stained with the ABL probe (scale bars = 100 µm) are shown (*n* = 81). G) The distribution of the difference in the ABL score (IRS) (△IRS = IRS_T_−IRS_N_) in cohort 1. H) Kaplan–Meier OS curves based on ABL expression in patients with GC in cohort 1 (*n* = 81). I) Time‐dependent receiver operating characteristic (ROC) curve analysis of the clinical risk score (TNM stage), ABL risk score, and combined ABL and clinical risk score in cohort 1. J) The distribution of the difference in the ABL score (IRS) (△IRS = IRS_T_−IRS_N_) in cohort 2 (*n* = 192). K) Kaplan–Meier OS curves based on ABL expression in patients with GC in cohort 2. L) Time‐dependent ROC curve analysis of the clinical risk score (TNM stage), the ABL risk score, and the combined ABL and clinical risk score in cohort 2. M,N) Multivariate analyses were performed for GC cohorts 1 and 2. All bars correspond to 95% CIs. AUC, area under the curve; CI, confidence interval. GC, gastric cancer; HR, hazard ratio; OS, overall survival; TNM, tumor, node, metastasis. The differences in IRS for ABL staining in primary tumors and corresponding normal tissues were assessed by the Wilcoxon test (grouped) (G and J). The probability of differences in OS was ascertained by the Kaplan‐Meier method with the log‐rank test (H and K). The data were analyzed by a two‐tailed unpaired Student's *t*‐test (C and D). The data are represented as the means ± SEM. * *p* < 0.05; ** *p* < 0.01; *** *p* < 0.001, NS, no significance.

To profile the lncRNAs expression in GC, we first analyzed four pairs of cancerous and matched adjacent normal tissue specimens from GC patients through RNA sequencing (RNA‐seq). Representative upregulated and downregulated lncRNAs (fold change >2 or <1/2, *p*‐value < 0.05) are shown in the heatmap (**Figure** **1**A and Table S1, Supporting Information). Intriguingly, two lncRNAs named RP3‐512B11.3 (ABL) and LOC730102 were identified as the top hits for further study (Figure 1B). Next, we analyzed the associations between selected lncRNA expression and GC prognosis using data from The Cancer Genome Atlas (TCGA) and the bioinformatics tool GEPIA (http://gepia.cancer‐pku.cn/), results of which showed that GC patients with higher ABL expression had worse outcomes (*p* = 0.024), while LOC730102 expression was not significantly associated with GC prognosis (*p* = 0.61) (Figure S1A,B, Supporting Information). Therefore, the lncRNA ABL was selected as the candidate for further study.

A 5′‐ and 3′‐ rapid amplification of cDNA ends (RACE) assay was used to determine the entire sequence of ABL, confirming that the sequence detected in BGC‐823 cells is identical to the transcript of ABL (*ENST00000561592*) archived in the UCSC database (Figure S1C, Supporting Information, http://genome.ucsc.edu/). In addition, computational analyses and the 3′‐Flag tagging strategy confirmed that ABL had no coding capacity (Figure S1D,E, Supporting Information). Subsequently, the expression of ABL in GC tissues was determined by qRT‐PCR, indicating that ABL expression was significantly upregulated in cancerous GC tissues compared with the paired adjacent normal tissues (Figure 1C). In addition, the abundance of ABL was significantly increased in GC cell lines compared with human normal gastric mucosal tissues (hGT) (Figure S1F, Supporting Information). RNAscope and RNA fluorescence in situ hybridization (RNA‐FISH) assays were performed and confirmed the significant abundance of ABL in GC cells compared to paired normal gastric mucosal cells in GC tissues (Figure 1D,E). To further investigate the clinical relevance of ABL expression in GC, we performed an RNA‐FISH assay with a tissue microarray of cohort 1, which showed that the expression of ABL was much higher in cancerous GC tissues than in matched normal gastric tissues (*n* = 81, *p* = 0.024; Figure 1F,G). Moreover, Kaplan–Meier analysis indicated that GC patients with higher expression of ABL had reduced overall survival (*n* = 81, *p* = 0.02968, log‐rank test; Figure 1H). Furthermore, univariate Cox regression analysis revealed that the tumor (T), node (N), and metastasis (M) categories and ABL expression were substantially associated with the survival in GC patients (Figure S1G, Supporting Information), and multivariate Cox regression analysis indicated that ABL expression was an independent predictive marker for the prognosis of patients with GC (Figure 1M). To further evaluate the predictive value of ABL expression, we conducted a time‐dependent receiver operating characteristic curve analysis. The combination of the clinical risk score (TNM stage) and ABL risk score was more accurate than either score alone in cohort 1 (Figure 1I). For example, the area under the curve (AUC) at year 5 was 0.712 for the clinical risk score, whereas it was significantly increased to 0.889 for the combination of the clinical risk score and ABL risk score. Furthermore, these results in cohort 1 were also supported by an independent cohort with more GC patients (cohort 2, *n* = 192), which suggested that ABL expression was significantly increased in GC patients with a poor prognosis (Figure 1J,K). Moreover, univariate/multivariate Cox regression analyses also revealed that the T, N, and M categories and ABL expression were substantially associated with survival in patients with GC (Figure 1N and Figure S1H, Supporting Information). The AUC also indicated that the combination of TNM stage and the ABL risk score was more accurate than either score alone in cohort 2 (Figure 1L). Taken together, these data reveal that ABL expression is significantly increased in GC and that ABL may be an independent prognostic factor for GC patients.

### 2.2 ABL Directly Binds to APAF1 via Its WD1/WD2 Domain

**Figure 2:**
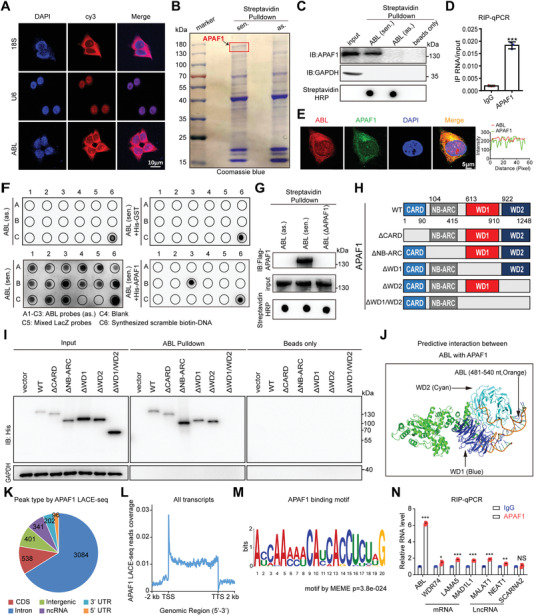
ABL directly binds to APAF1. A) Localization of ABL in BGC823 cells detected by RNA‐FISH. U6 and 18S rRNA were used as positive controls for the nuclear and cytoplasmic fractions, respectively. B) Coomassie brilliant blue staining of proteins pulled down by biotinylated ABL. Sen., sense transcript; as., antisense transcript. C) Western blot detection of APAF1 pulled down by in vitro‐transcribed biotinylated ABL from BGC‐823 cell lysates. GAPDH was used as a negative control. D) The interaction of ABL with APAF1 in BGC823 cells was shown by RIP‐qPCR detection of the ABL pulled down by an anti‐APAF1 antibody. E) Confocal images showing colocalization of ABL (red) and APAF1 (green) in BGC823 cells (scale bars = 5 µm, left panel). Right panel: the images were subject to *Z*‐axis profile analysis. F) In vitro RNA pull‐down coupled with a dot blot assay using the indicated RNA transcripts and recombinant APAF1 proteins. Bottom panel: Annotation of each dot. G) In vitro‐transcribed antisense, sense, or sense with deletion of nt 481–540 (binding region for APAF1) ABL transcripts were incubated with recombinant histidine (His)‐tagged APAF1 proteins for an in vitro streptavidin RNA pull‐down assay, followed by Western blot detection using an anti‐His antibody. H) Graphic illustration of APAF1 deletion mutants. I) In vitro RNA protein binding assay showing the interaction of biotinylated ABL with Flag‐tagged APAF1 proteins, including the WT protein and CARD, NB‐ARC, WD1, WD2, or WD1/WD2 deletion mutants. J) Docking analysis of the protein‐RNA interaction model between APAF1 and ABL (nt 481–540). K) Genomic distribution of the APAF1 binding peaks from LACE‐seq reads. L) The meta profile of APAF1‐RNA interacting sites. TSS: transcription start site; TTS: transcription termination site. M) Enriched sequence among APAF1‐RNA crosslinking sites by the Multiple Em for Motif Elicitation (MEME) tool. N) RIP‐qPCR analysis was used to detect whether APAF1 could enrich the mRNAs or lncRNAs identified in LACE‐seq. The data were analyzed by a two‐tailed unpaired Student's *t*‐test (D and N). The data are represented as the means ± SEM of three independent experiments. * *p* < 0.05; ** *p* < 0.01; *** *p* < 0.001, NS, no significance.

To elucidate the functional roles of ABL in GC, we first examined its subcellular localization. An RNA‐FISH assay showed that ABL was predominantly located in the cytoplasm (**Figure** **2**A). To identify the protein partners of ABL, in vitro‐transcribed biotinylated ABL sense and antisense transcripts were subjected to lncRNA pull‐down followed by mass spectrometry (MS). Notably, the sense ABL transcript was associated specifically with APAF1 (Figure 2B and Table S2, Supporting Information). Then, RNA pull‐down assays performed with cell lysates and RNA immunoprecipitation (RIP) assays further confirmed the specific association of ABL with APAF1 (Figure 2C,D and Figure S2A, Supporting Information). Furthermore, RNA‐FISH combined with immunofluorescence (IF) showed a cytoplasmic colocalization pattern for ABL and APAF1 (Figure 2E). To identify the ABL sequence motifs responsible for APAF1 binding, we performed in vitro RNA pull‐down followed by a dot blot assay as described previously.^[^ ^10b^ ^]^ The motif sequence of ABL bound by APAF1 was identified to encompass nt 481–540 (Figure 2F and Figure S2B, Supporting Information). Furthermore, an in vitro RNA‐protein binding assay using recombinant APAF1 also confirmed that ABL could directly interact with APAF1, but deletion of the corresponding sequence (nt 481–540) in ABL completely abolished its interaction with APAF1 (Figure 2G and Figure S2C, Supporting Information). In addition, protein domain mapping analysis demonstrated that ABL bound to the region containing amino acids (aa) 613–1248 at the C terminus of APAF1 containing 15 WD40 repeats, which constitute two *β*‐propellers named WD1 (aa 613–910) and WD2 (aa 922–1248) (Figure 2H,I). The WD40 domain is a noncanonical RNA‐binding motif,^[^ ^9b^ ^]^ which is consistent with our finding that the WD1/WD2 domain of APAF1 serves as the RNA‐binding domain for ABL. Intriguingly, RNA–protein docking analysis also showed that the nt 481–540 region of ABL perfectly interacted with the WD1/WD2 domain of APAF1 using the PDB2PQR server (Figure 2J and Figure S2D,E, Supporting Information). Based on these results, we speculated that APAF1 may be an RNA binding protein (RBP), we next performed the linear amplification of complementary DNA ends and sequencing (LACE‐seq)^[^ ^12^ ^]^ to confirm this hypothesis. APAF1‐binding sites identified by LACE‐seq were mapped to 2493 transcripts (4662 binding peaks), which were distributed in the coding sequence (CDS), intergenic regions, intronic regions, noncoding RNA (ncRNA), 3′ untranslated region (UTR), and 5′ UTR (Figure 2K and Figure S2F, Supporting Information). Moreover, we found that APAF1 showed enriched binding around the transcription start site (TSS) (Figure 2L). Meanwhile, the APAF1 binding motif was analyzed by the Multiple Em for Motif Elicitation tool (MEME) (Figure 2M), and the “AACCUU_AG” consense sequence could be mapped in the 481–540 nt of ABL transcript, which was interacted with the WD1/WD2 domain of APAF1. Then RIP‐qPCR assay confirmed that APAF1 could significantly interact with mRNAs or lncRNAs identified in LACE‐seq. Among them, ABL was more enriched than other RNAs (Figure 2N). Meanwhile, overexpression of ABL resulted in reduced binding of APAF1 to other RNAs (Figure S2G, Supporting Information). Collectively, these results indicate that ABL could directly interact with APAF1 as a potential RBP.

### 2.3 ABL Promotes GC Cell Survival and Inhibits Apoptosis Induction by Multiple Drugs

**Figure 3:**
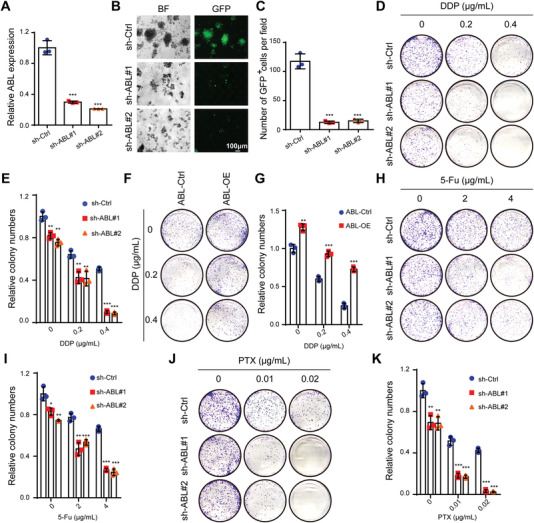
Knockdown of ABL promotes GC cell apoptosis and sensitivity to multiple drugs in vitro. A) Knockdown efficiencies were verified in MKN45 cells by qRT‐PCR. B) Suspension growth assays were used to determine the cell survival of ABL‐deficient MKN45 cells. C) Quantification of the GFP^+^ cells in (B). D) Knockdown of ABL decreased the colony‐forming ability of MKN45 cells and promoted DDP‐induced apoptosis in these cells. E) Quantification of the colony formation assay results in (D). F) Overexpression of ABL increased the colony‐forming ability of BGC823 cells and antagonized DDP‐induced apoptosis in these cells. G) Quantification of the colony formation assay results in (F). H) Knockdown of ABL decreased the colony‐forming ability of MKN45 cells and promoted 5‐Fu‐induced apoptosis in these cells. I) Quantification of the colony formation assay results in (H). J) Knockdown of ABL decreased the colony‐forming ability of MKN45 cells and promoted PTX‐induced apoptosis in these cells. K) Quantification of the colony formation assay results in (J). The data were analyzed by a two‐tailed unpaired Student's *t*‐test (A, C, E, G, I, and K). The data are represented as the means ± SEM of three independent experiments. * *p* < 0.05; ** *p* < 0.01; *** *p* < 0.001, NS, no significance.

APAF1 is essential for intrinsic pathway‐mediated cell death, which is induced by developmental lineage information, oncogene activation, DNA damage, and nutrient deprivation,^[^ ^7b^ ^]^ and is associated with chemoresistance.^[^ ^13^ ^]^ Therefore, we speculated that ABL modulates this process by binding to APAF1. First, we generated stable ABL‐knockdown and ABL‐overexpressing GC cells (**Figure** **3**A and Figure S3A, Supporting Information). Knockdown of ABL significantly reduced GC cell growth, while overexpression of ABL promoted GC cell growth, as determined by a CCK‐8 assay (Figure S3B,C, Supporting Information). Furthermore, knockdown of ABL obviously suppressed GC cell growth in ultralow‐attachment culture plates, while upregulation of ABL promoted cell growth (Figure 3B,C and Figure S3D,E, Supporting Information). Then, a colony formation assay showed that the colony numbers of ABL‐deficient cells were significantly decreased but those of ABL‐overexpressing cells were increased compared to the colony numbers of the corresponding control cells (Figure 3D–G). In addition, when cells were treated with cisplatin (DDP) at the indicated doses, the colony numbers of ABL‐deficient cells were much more significantly decreased than those of control cells (Figure 3D,E). Conversely, ABL‐overexpressing cells showed significantly increased colony numbers compared to control cells after cisplatin treatment (Figure 3F,G). We further assessed this effect of ABL with 5‐Fu and PTX treatments. The results also supported the conclusion that knockdown of ABL accelerated 5‐Fu‐ and PTX‐induced apoptosis, while overexpression of ABL promoted cell survival with or without 5‐Fu or PTX treatment (Figure 3H–K and Figure S3F–I, Supporting Information). Taken together, these data reveal that ABL promotes GC cell survival and may be involved in multidrug resistance.

### 2.4 ABL Antagonizes GC Cell Apoptosis by Competitively Blocking the Interaction of Cyt c with APAF1

**Figure 4:**
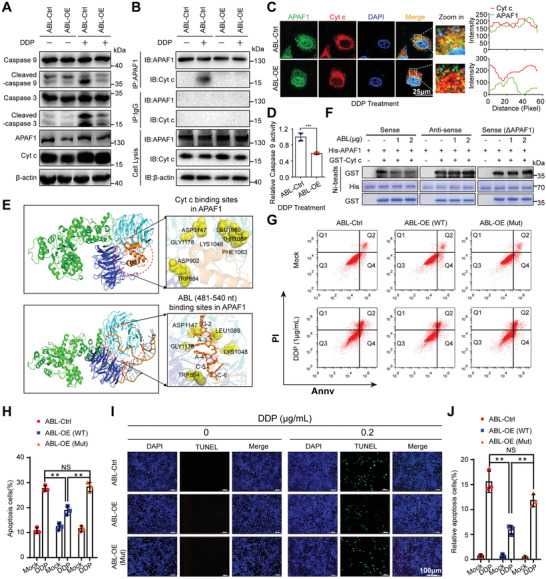
ABL antagonizes GC cell apoptosis by competitively blocking the binding of APAF1 with Cyt c. A) Western blotting was applied to determine the expression of the indicated proteins in ABL‐overexpressing BGC823 cells after DDP treatment at 0 or 1 µg mL^−1^ for 24 h. B) Immunoprecipitation (IP) and Western blotting were used to detect the interaction between APAF1 and Cyt c in ABL‐overexpressing BGC823 cells after DDP treatment at 0 or 1 µg mL^−1^ for 24 h. C) Confocal images showing colocalization of APAF1 (green) and Cyt c (red) in ABL‐overexpressing BGC823 cells after DDP treatment at 1 µg mL^−1^ for 24 h (scale bars = 25 µm, left panel). Right panel: the images were subject to *Z*‐axis profile analysis. D) Caspase‐9 activity was detected in ABL‐overexpressing BGC823 cells and corresponding control cells after DDP treatment at 1 µg mL^−1^ for 24 h. E) Docking analysis of the protein–protein or protein–RNA interaction model between APAF1‐Cyt c and APAF1‐ABL (nt 481–540), respectively. F) His‐tagged WD1/WD2 domain of APAF1 bound to Ni‐NTA beads was incubated with or without increasing amounts of 1 or 2 µg ABL sense, anti‐sense, or mutant sense (∆APAF1) and purified GST‐Cyt c. Bead‐bound proteins and the input were analyzed by western blot and coomassie blue staining. G) An annexin‐V‐FITC/PI assay was used to detect apoptotic ABL‐overexpressing BGC823 cells after DDP treatment at 0 or 1 µg mL^−1^ for 24 h. H) Quantification of the apoptotic cells in (G). I) A TUNEL assay was used to detect apoptotic ABL‐overexpressing and corresponding control BGC823 cells after DDP treatment at 0 or 1 µg mL^−1^ for 24 h. J) Quantification of the apoptotic cells in (I). The data were analyzed by a two‐tailed unpaired Student's *t*‐test (D, H, and J). The data are represented as the means ± SEM of three independent experiments. * *p* < 0.05; ** *p* < 0.01; *** *p* < 0.001, NS, no significance.

As multidrug resistance is often correlated to ABC transporters such as P‐gp/ABCB1, MRP1/ABCC1, and/or BCRP/ABCG2;^[^ ^14^ ^]^ however, the protein levels of them were not obviously affected in ABL‐overexpressing and ABL‐deficient cells with or without DDP or PTX treatment (Figure S3J, Supporting Information). To further characterize the mechanism by which ABL modulates cell apoptosis and chemoresistance, we first detected the protein levels of key molecules involved in the intrinsic apoptosis pathway. The upregulation of ABL decreased the cisplatin‐induced expression of cleaved caspase‐9/3, but the knockdown of ABL increased this expression (**Figure** **4**A and Figure S4A, Supporting Information). However, the protein levels of APAF1 and Cyt c were not obviously affected (Figure 4A and Figure S4A, Supporting Information). Considering that Cyt c is sandwiched between the two front faces of WD1 and WD2 of APAF1,^[^ ^15^ ^]^ which is also the ABL‐binding domain, we asked whether ABL binding to APAF1 competitively blocks the interaction of Cyt c with APAF1. Indeed, the ability of APAF1 to bind to Cyt c was dramatically inhibited in ABL‐overexpressing GC cells treated with cisplatin (Figure 4B). In addition, we found that the colocalization of APAF1 and Cyt c in ABL‐overexpressing cells treated with cisplatin was decreased compared to that in control cells (Figure 4C). Moreover, overexpression of ABL significantly inhibited cisplatin‐induced caspase‐9 activation (Figure 4D). Intriguingly, ABL‐APAF1 structure docking analysis also indicated that the region including nt 481–540, especially the ^482^GAACC^486^ motif, in ABL was located in close proximity to Leu1089/Gly1178 in the WD2 domain and Trp884 in the WD1 domain of APAF1 (Figure 4E and Figure S4B, Supporting Information), which are also the specific residues for the APAF1‐Cyt c interaction determined by cryo‐electron microscopy.^[^ ^15^ ^]^ Next, to further determine whether ABL could block the binding of Cyt c to APAF1, in vitro‐transcribed ABL sense, antisense, and mutant sense (∆APAF1, deletion of APAF1‐binding sites, nt 481–540) transcripts were subjected to incubate with purified APAF1‐WD1/WD2 domain and Cyt c protein, which showed that the amount of Cyt c bound to the WD1/WD2 domain of APAF1 was drastically decreased in the presence of ABL sense, but not ABL antisense or mutant sense (∆APAF1) (Figure 4F). Collectively, these data indicate that ABL competitively inhibits the interaction of Cyt c with APAF1 and thus impairs the activation of caspase‐9.

Subsequently, we examined whether ABL antagonizes GC apoptosis in a manner dependent on the core region of nt 481–540. Plasmids expressing wild‐type ABL (WT) or mutant ABL (deletion of nt 481–540, Mut) were constructed. The results of an Annexin‐V‐FITC/PI staining assay showed that overexpression of ABL‐WT but not ABL‐Mut reduced cisplatin‐induced apoptosis compared to control expression (Figure 4G,H). The results were also confirmed by a TUNEL assay for the detection of apoptotic cells (Figure 4I,J). In contrast, knockdown of ABL dramatically promoted cisplatin‐induced GC cell apoptosis (Figure S4C–F, Supporting Information).

To further confirm the role of ABL antagonizes apoptosis by competitively blocking the interaction of Cyt c with APAF1, we generated APAF1 knockout BGC823 cells through the CRISPR/Cas9 gene‐editing system (Figure S4G, Supporting Information). APAF1‐knockout cells showed significantly increased colony numbers and antagonized the effect of cisplatin compared to control cells (Figure S4H,I, Supporting Information). Meanwhile, overexpression of either ABL‐WT or ABL‐Mut in APAF1 knockout cells showed no obvious antagonistic effect of cisplatin compared to the control cells (Figure S4J,K, Supporting Information).

**Figure 5:**
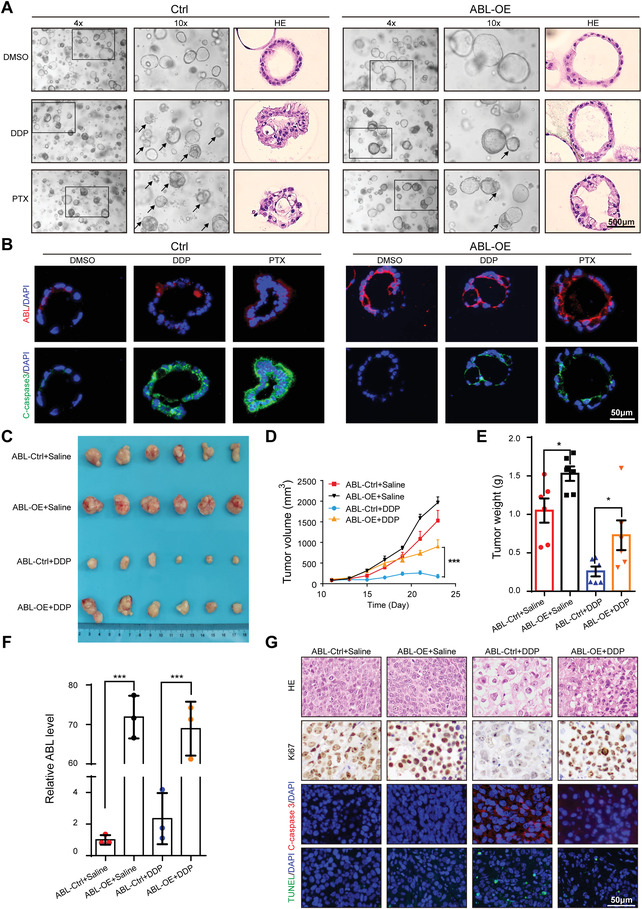
ABL promotes GC cell growth and multidrug resistance in vivo. A) GC organoids were infected with ABL overexpression vectors or a control lentivirus and treated with or without DDP (5 µg mL^−1^) or PTX (0.4 µg mL^−1^) for 24 h. Representative bright‐field images and hematoxylin and eosin (H&E) staining are shown (scale bars = 500 µm, *n* = 3). B) Sections of organoids were stained with ABL probes and an anti‐cleaved caspase‐3 antibody (scale bars = 50 µm). C) Overexpression of ABL effectively promoted subcutaneous GC tumor growth and antagonized the antitumor effect of cisplatin in nude mice (*n* = 6). D) Tumor volume was monitored every other day, and tumor growth curves were generated. E) Tumors were extracted and weighed at the end of the experiment. F) The expression of ABL was detected in each group by qRT‐PCR. G) Sections of tumors were stained with H&E, and anti‐Ki67 antibody, an anti‐cleaved caspase‐3 antibody, or TUNEL reagents (scale bars = 50 µm). The data were analyzed by one‐way ANOVA test followed by Turkey's multiple comparisons (D). The data were analyzed by a two‐tailed unpaired Student's *t*‐test (E and F). The data are represented as the means ± SEM of three independent experiments. * *p* < 0.05; ** *p* < 0.01; *** *p* < 0.001, NS, no significance.

To further examine the roles of ABL in drug‐induced apoptosis, we also established a GC organoid model. Experimental results showed that overexpression of ABL by lentiviral infection significantly promoted GC cell growth in GC organoids and that the diameter of ABL‐overexpressing tumor organoids was significantly increased compared with that of their counterparts (Figure S4L, Supporting Information). Furthermore, when organoids were treated with cisplatin or PTX, the organoids with upregulation of ABL showed significantly reduced cisplatin/PTX‐induced apoptosis compared to the corresponding control organoids (**Figure** **5**A). Cleaved caspase‐3 staining also indicated that the number of apoptotic cells decreased dramatically in the ABL‐overexpressing group compared with the control group (Figure 5B). These results were also validated by a TUNEL assay (Figure S4M, Supporting Information). Then, we performed tumor xenograft studies to verify the roles of ABL in GC growth and cisplatin sensitivity in vivo. The results showed that overexpression of ABL could promote tumor growth, as demonstrated by the size and weight of tumors in the overexpression group compared with those in the control group (Figure 5C–E). Moreover, overexpression of ABL significantly antagonized the antitumor effect of cisplatin (Figure 5C–E). The expression of ABL was determined to be significantly increased in the ABL‐overexpressing groups compared to the control groups (Figure 5F). Additionally, immunohistochemistry (IHC) results showed increased expression of Ki‐67, a marker of proliferation, in the tumor tissues of the ABL‐overexpressing group compared with that of the control group with or without cisplatin treatment (Figure 5G). Finally, cleaved caspase‐3 staining and TUNEL assays showed that the number of apoptotic cells was significantly decreased in the ABL‐overexpressing group compared with the control group after cisplatin treatment (Figure 5G). These results suggest that ABL antagonizes drug‐induced apoptosis by competitively blocking the binding of Cyt c with APAF1.

### 2.5 IGF2BP1 Binds and Recognizes the METTL3‐Mediated m6A Modification on ABL, Maintaining ABL Stability

**Figure 6:**
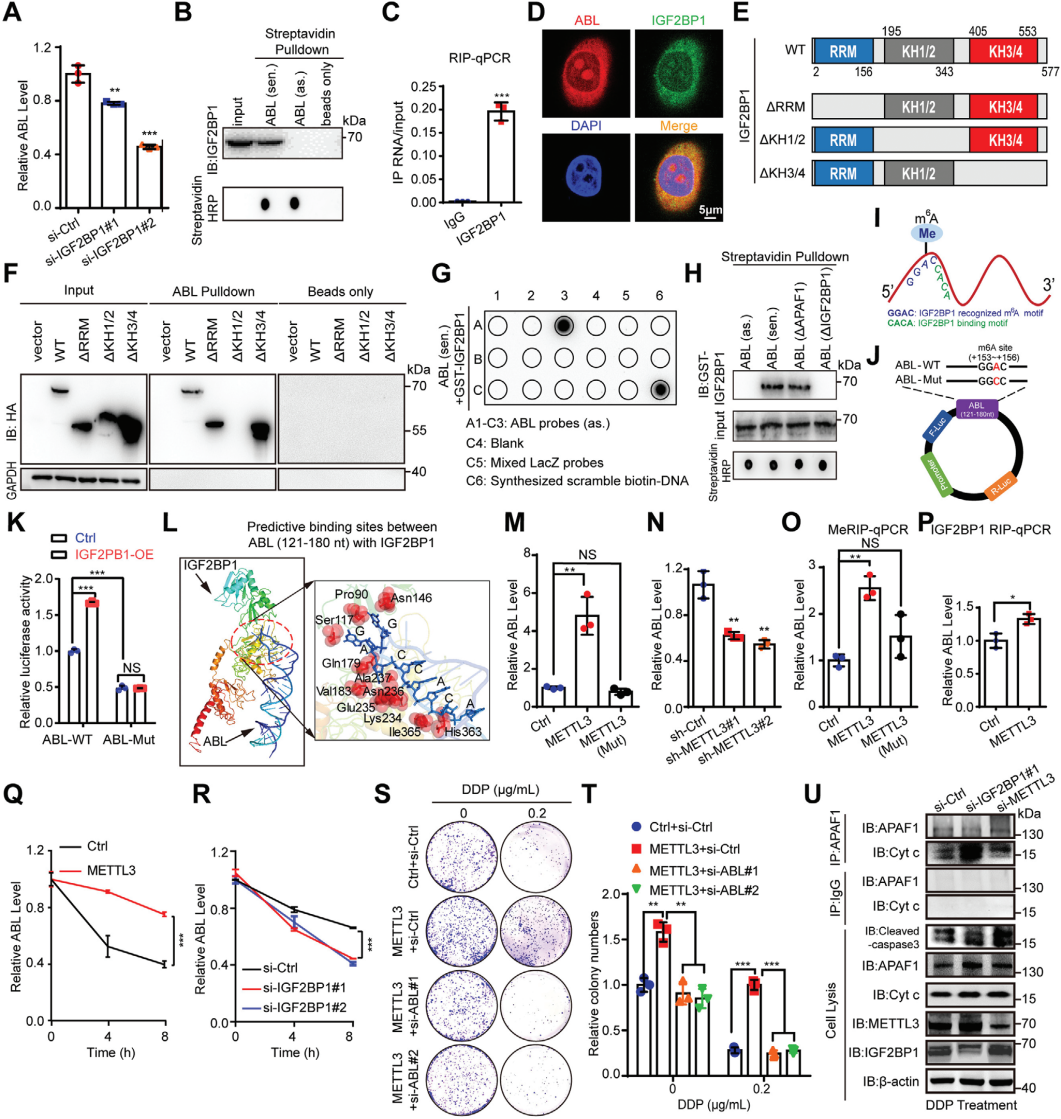
IGF2BP1 binds and recognizes the METTL3‐mediated m^6^A modification on ABL, maintaining ABL stability. A) The ABL levels in BGC823 cells with IGF2BP1 deficiency were determined by qRT‐PCR. B) Western blot detection of IGF2BP1 pulled down by in vitro‐transcribed biotinylated ABL from BGC‐823 cell lysates. Sen., sense transcript; as., antisense transcript. C) The interaction of ABL with IGF2BP1 in BGC823 cells was examined by a RIP‐qPCR assay. D) Confocal images showing colocalization of ABL (red) and IGF2BP1 (green) in BGC823 cells (scale bars = 5 µm). E) Graphic illustration of IGF2BP1 deletion mutants. F) In vitro RNA protein binding assay showing the interaction of biotinylated ABL with HA‐tagged IGF2BP1 proteins, including the WT protein and RRM, KH1/2, or KH3/4 deletion mutants. G) In vitro RNA pull‐down coupled with a dot blot assay using the indicated RNA transcripts and recombinant IGF2BP1 proteins. Bottom panel: Annotation of each dot. H) In vitro‐transcribed antisense, sense, sense with deletion of nt 121–180 (binding region for IGF2BP1) or sense with deletion of nt 481–540 (binding region for APAF1) ABL transcripts were incubated with recombinant His‐IGF2BP1 proteins for an in vitro streptavidin RNA pull‐down assay, followed by Western blot detection using an anti‐His antibody. I) Graphic illustration of the “CACA” motif in ABL for IGF2BP1 binding and the “GGAC” motif, including the m^6^A modification site for IGF2BP1 recognition. J) Schematic presentation of the construction of the luciferase reporter containing ABL‐WT or ABL‐Mut region. K) Relative luciferase activity of the ABL‐WT or ABL‐Mut luciferase reporter in BGC823 cells with IGF2BP1 overexpression and corresponding control cells was detected. L) The calculated protein–RNA interaction model between IGF2BP1 and ABL (nt 121–180). M,N) The ABL levels in GC cells with wild‐type or catalytic mutant (Mut) METTL3 overexpression and METTL3‐deficient were detected by qRT‐PCR. O) MeRIP‐qPCR analysis was used to demonstrate METTL3‐mediated m^6^A modifications on ABL. The m^6^A modification of ABL was increased upon upregulation of wide type METTL3 while no significant change upon of Mut METTL3. P) RIP‐qPCR analysis was used to demonstrate that IGF2BP1 could enrich more ABL upon overexpression of METTL3. Q) The levels of ABL expression in METTL3‐overexpressing and corresponding control GC cells treated with actinomycin D (2 µg mL^−1^) at the indicated time points were detected by qRT‐PCR. R) The levels of ABL expression in IGF2BP1‐deficient and corresponding control GC cells treated with actinomycin D (2 µg mL^−1^) at the indicated time points were detected by qRT‐PCR. S) Representative images of the cell colony formation abilities of METTL3‐overexpressing BGC823 cells transfected with ABL‐specific siRNAs or corresponding controls and treated with DDP at the indicated doses for 24 h. T) Quantification of the colony formation assay results in (S). U) Immunoprecipitation (IP) and Western blotting were used to detect the interaction between APAF1 and Cyt c in IGF2BP1 or METTL3 deficient BGC823 cells after DDP treatment at 1 µg mL^−1^ for 24 h. The data were analyzed by a two‐tailed unpaired Student's *t*‐test (A, C, K, M–R, and T). The data are represented as the means ± SEM of three independent experiments. * *p* < 0.05; ** *p* < 0.01; *** *p* < 0.001, NS, no significance.

To explore the mechanism of high expression of ABL in GC, we first analyzed the epigenetic modification in the promoter region of ABL with the UCSC genome browser (http://genome.ucsc.edu/). As shown in Figure S5A (Supporting Information), highly enriched H3K27 acetylation (H3K27ac) signals were found in the promoter region of ABL, suggesting that ABL expression may be activated by chromatin acetylation. H3K27ac is known to be catalyzed by the P300/CBP complex.^[^ ^16^ ^]^ However, P300 knockdown using two specific siRNAs did not regulate the level of ABL (Figure S5B,C, Supporting Information). Thus, we speculate that other mechanisms regulate the expression of ABL.

m^6^A methylation has been identified as the most abundant modification ubiquitously occurring in mRNAs and noncoding RNAs and can affect RNA stability, splicing, localization, and translation.^[^ ^17^ ^]^ Recent studies have reported that IGF2BPs, including IGF2BP1/2/3, are a distinct family of m^6^A readers that target thousands of RNAs to enhance RNA stability.^[^ ^18^ ^]^ Our previous study also showed that IGF2BP3‐dependent m^6^A modification maintains HDGF mRNA stability.^[^ ^19^ ^]^ Interestingly, MS results following biotinylated ABL pull‐down indicated that IGF2BP1/3 might bind to ABL (Table S2 and Figure S5D, Supporting Information), which inspired us to speculate that m^6^A modification might be associated with high ABL expression in GC. We first knocked down IGF2BP1 and IGF2BP3 using two specific siRNAs (Figure S5E,F, Supporting Information). Only knockdown of IGF2BP1 markedly suppressed ABL expression, while knocking down IGF2BP3 had no noticeable effect (**Figure** **6**A and Figure S5G, Supporting Information). In addition, RNA pull‐down and RIP assays confirmed the interaction of ABL with IGF2BP1 (Figure 6B,C). IF assay also indicated that cytoplasmic ABL co‐localized with IGF2BP1 (Figure 6D). Analysis of gene expression data from the GEPIA database (http://gepia.cancer‐pku.cn/) revealed that IGF2BP1 expression was significantly correlated with ABL expression (*R* = 0.23, *p* = 2.3 × 10^−6^, Figure S5H, Supporting Information). Furthermore, protein domain mapping analysis showed that ABL specifically bound to the KH1/2 domain of IGF2BP1 (Figure 6E,F). We also found that the motif sequence of ABL bound/protected by IGF2BP1 was identified to encompass nt 121–180 (Figure 6G and Figure S2B, Supporting Information). Deletion of this sequence in ABL (nt 121–180) completely abolished its direct interaction with recombinant IGF2BP1, whereas deletion of the APAF1‐binding sequence (nt 481–540) in ABL did not affect this binding (Figure 6H and Figure S2C, Supporting Information). A previous study showed that IGF2BP1/3 preferentially interacts with CA‐rich sequences,^[^ ^20^ ^]^ and IGF2BPs can also recognize the key m^6^A motif GGAC.^[^ ^18^ ^]^ Surprisingly, we found the core sequence (nt 121–180) of ABL contains the “^122^GGACCACA^129^” motif, implying that IGF2BP1 may directly bind to the “CACA” motif and then recognize the m^6^A motif “GGAC” of ABL (Figure 6I). To further verify the “GGAC” motif in the 121–180 nt region of ABL was modified by m6A, we replaced the adenosine (A) with cytosine (C) and inserted a partial 121–180 nt sequence of ABL with a wild‐type or mutated m6A site into luciferase reporter plasmids (Figure 6J), which demonstrated that IGF2BP1 caused upregulation of the relative luciferase activity in ABL‐WT group, but not in ABL‐Mut group (Figure 6K). Furthermore, RNA‐protein docking analysis also showed that the “GGACCACA” sequence of ABL played an essential role in binding to several residues in the KH1/2 domain of IGF2BP1 (Figure 6L).

METTL3 was first identified as the major catalytic enzyme (writer) that catalyzes m^6^A modification.^[^ ^17b^ ^]^ To further explore the effects of m^6^A on ABL regulation, the plasmids expressing wild‐type METTL3 or its catalytic mutant (aa395‐398, DPPW→APPA) were constructed following our previous study,^[^ ^19^ ^]^ and the m^6^A level was dramatically decreased in BGC823 cells with catalytic mutant METTL3 compared with wild type cells (Figure S5I, Supporting Information). We also established stable METTL3‐knockdown GC cells (Figure S5J, Supporting Information). The results revealed that the expression of ABL was increased in METTL3‐overexpressing cells while catalytic mutant METTL3 overexpression could not boost ABL expression (Figure 6M). Meanwhile, the knockdown of METTL3 reduced the level of ABL (Figure 6N). Analysis of gene expression data showed that the expression of METTL3 was positively correlated with ABL expression (*R* = 0.11, *p* = 0.024, Figure S5K, Supporting Information, http://gepia.cancer‐pku.cn/). MeRIP‐qPCR data also showed that an m^6^A‐specific antibody significantly enriched ABL upon wide type METTL3 overexpression; however, it had no obvious effect in catalytic mutant METTL3‐overexpressing cells compared to the control cells (Figure 6O). Interestingly, we found that IGF2BP1 could enrich more ABL upon overexpression of METTL3 through a RIP‐qPCR assay (Figure 6P), indicating that the m^6^A modification on ABL facilitated its binding to IGF2BP1. Subsequently, we explored the effect of IGF2BP1 on ABL stability. GC cells were treated with actinomycin D, an inhibitor of transcription, for the indicated times. The level of ABL was shown to be highly stable upon METTL3 overexpression, while the opposite effect was observed upon knockdown of IGF2BP1 (Figure 6Q,R).

To further characterize the oncogenic function of the IGF2BP1‐ABL axis in GC cell survival and drug resistance, a colony formation assay was performed. As expected, knockdown of IGF2BP1 markedly inhibited GC cell survival and promoted cisplatin‐induced apoptosis (Figure S5L,M, Supporting Information). Furthermore, overexpression of IGF2BP1 significantly promoted GC cell survival and counteracted cisplatin‐induced apoptosis, but knockdown of ABL in IGF2BP1‐overexpressing GC cells using specific siRNAs markedly suppressed these effects (Figure S5N–P, Supporting Information). Moreover, overexpression of METTL3 significantly promoted GC cell survival and counteracted cisplatin‐induced apoptosis, but knockdown of ABL in METTL3‐overexpressing GC cells using specific siRNAs markedly suppressed these effects (Figure 6S,T and Figure S5Q, Supporting Information). Furthermore, knockdown of IGF2BP1 or METTL3 increased the cleaved caspase‐3 expression and the ability of APAF1 to bind to Cyt c after cisplatin treatment (Figure 6U). Thus, our data suggest that IGF2BP1 binds to and recognizes the METTL3‐mediated m^6^A modification on ABL, and stabilizes ABL transcripts, which may lead to multidrug resistance.

### 2.6 Liposome‐Mediated Codelivery of ABL‐Specific siRNA and PTX Synergistically Induce GC Cell Apoptosis In Vivo

**Figure 7:**
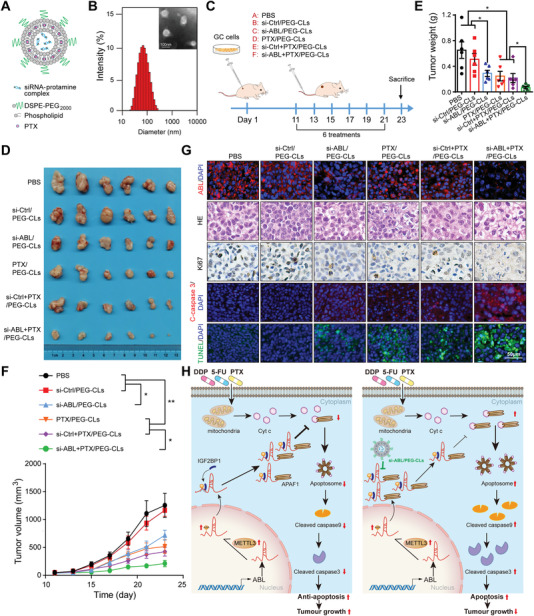
Nanoencapsulated ABL‐specific siRNA and PTX synergistically induce GC cell apoptosis in vivo. A) Graphic illustration of si‐ABL and PTX encapsulated in PEG‐CLs. B) Representative TEM image and the size distribution profile of si‐ABL+PTX/PEG‐CLs. C) Graphic illustration of the therapeutic model. D) ABL‐specific siRNA‐loaded PEG‐CLs inhibited subcutaneous GC tumor growth and promoted PTX‐induced apoptosis in nude mice (*n* = 6). E) Tumors were extracted and weighed at the end of the experiment. F) Tumor volume was monitored every other day, and tumor growth curves were generated. G) Sections of tumors were stained with an ABL probe, hematoxylin and eosin (H&E), an anti‐Ki67 antibody, an anti‐cleaved caspase‐3 antibody, or TUNEL reagents (scale bars = 50 µm). H) Graphic illustration of ABL modulating GC cell apoptosis induced by chemotherapeutic drugs and knockdown of ABL (using nanoencapsulated siRNA) and nanoencapsulated PTX synergistically inducing GC cell apoptosis. The data were analyzed by a two‐tailed unpaired Student's *t*‐test (E). The data were analyzed by one‐way ANOVA test followed by Turkey's multiple comparisons (F). The data are represented as the means ± SEM of three independent experiments. * *p* < 0.05; ** *p* < 0.01; *** *p* < 0.001, NS, no significance.

To investigate the potential value of ABL as a therapeutic target in GC, we developed poly (ethylene glycol)‐modified cationic liposomes (PEG‐CLs) loaded with ABL‐specific siRNA. The anionic ABL‐specific siRNA (si‐ABL) was first condensed by cationic protamine, and the formed siRNA/protamine complex was further encapsulated in PTX‐loaded cationic liposome (**Figure** **7**A). The N:P ratio of the siRNA/protamine complex was 4:1, and the amount of total lipid was 100 nmol µg^−1^ (lipid/siRNA). The obtained CLs could efficiently encapsulate the siRNA molecules at the optimal N:P ratio and lipid amount (Figure S6A,B and Tables S3 and S4, Supporting Information). PEGylation was introduced to enhance the in vivo stability and circulation of the liposomes. si‐ABL/PEG‐CLs showed favorable in vitro gene silencing efficiency (Figure S6C, Supporting Information). An intracellular distribution assay showed that PEG‐CLs could deliver FAM‐siRNA into the cytoplasm effectively through endolysosomal escape after cellular uptake (Figure S6D, Supporting Information). Optimized si‐ABL and PTX coloaded PEG‐CLs (si‐ABL+PTX/PEG‐CLs) with a PEG to total lipids molar ratio of 5% had an average diameter of 73.12 ± 1.92 nm and a zeta potential of 0.172 ± 0.609 mV (Figure 7B, Figure S6E and Table S5, Supporting Information). A cellular uptake study confirmed that either FAM‐scrRNA or free PTX showed a low intracellular concentration in GC cells, while FAM‐scrRNA+PTX/PEG‐CLs and scrRNA+PTX/PEG‐CLs could be readily internalized by GC cells, supporting much higher intracellular amounts of scrRNA and PTX (Figure S6F,G, Supporting Information). We then performed a colony formation assay to assess the in vitro antitumor capability of si‐ABL+PTX/PEG‐CLs. The results showed that si‐ABL/PEG‐CLs, PTX/PEG‐CLs, and si‐Ctrl+PTX/PEG‐CLs inhibited GC survival compared with control treatment (Figure S6H,I, Supporting Information). Overall, the si‐ABL+PTX/PEG‐CL group showed a better in vitro antitumor capability than the other groups (Figure S6H,I, Supporting Information).

Furthermore, we evaluated the in vivo antitumor efficacy of si‐ABL+PTX/PEG‐CLs using a xenograft mouse model. Different liposome formulations were injected into the model mice via the tail vein at the indicated times (Figure 7C). The results showed that si‐ABL/PEG‐CLs, PTX/PEG‐CLs, and si‐Ctrl+PTX/PEG‐CLs could inhibit tumor growth, as reflected by the tumor size and weight, compared with PBS or si‐Ctrl/PEG‐CLs (Figure 7D–F). Among all the groups, the si‐ABL+PTX/PEG‐CL group showed the highest antitumor capability (Figure 7D–F). Moreover, the expression of ABL was confirmed to be significantly decreased in the si‐ABL/PEG‐CL and si‐ABL+PTX/PEG‐CL groups by an RNA‐FISH assay (Figure 7G). We also found that the number of apoptotic cells markedly increased, while that of Ki‐67‐positive cells decreased in mice treated with si‐ABL/PEG‐CLs, PTX/PEG‐CLs or si‐Ctrl+PTX/PEG‐CLs alone, and this effect was more obvious in the si‐ABL+PTX/PEG‐CL group than in the PBS or si‐Ctrl/PEG‐CL group (Figure 7G), indicating the synergistic antitumor capability of si‐ABL and PTX.

In addition, we evaluated systemic toxicity by evaluating the body weight of mice, and the data showed that the body weights of mice in different groups were not significantly different, indicating that si‐ABL and PTX at the doses and administration frequencies used had no systemic toxicity (Figure S7A, Supporting Information). Furthermore, we also evaluated tissue toxicity using hematoxylin and eosin (H&E) staining, which exhibited no significant toxicity to major organs, including the heart, liver, spleen, lungs, and kidneys (Figure S7B, Supporting Information). Collectively, these data suggest that knockdown of ABL may effectively increase the sensitivity of GC to chemotherapy.

## 3 Discussion

Emerging evidence has suggested lncRNAs as a new class of factors involved in the development and progression of cancer.^[^ ^21^ ^]^ In this study, we identified a new lncRNA, ABL, that was significantly upregulated in GC tissues and associated with a poor prognosis in GC patients. Functionally, IGF2BP1 directly bound and recognized the METTL3‐mediated m^6^A modification on ABL and maintained ABL stability. Thus, abundant ABL inhibited the intrinsic apoptosis pathway by competitively blocking the interaction of APAF1 with Cyt c and thus preventing assembly of the apoptosome in GC cells and subsequent activation of caspase‐9/3. Importantly, our study explored a novel therapeutic strategy targeting ABL using nanoencapsulated siRNA and coloaded PTX, which could significantly enhance the antitumor effect of PTX on GC (Figure 7H).

The mitochondrial apoptosis pathway is a common process by which the first‐line chemotherapeutic drugs induce cancer cell death.^[^ ^13c^ ^]^ Upon cell‐intrinsic stimulation, APAF1 binds to Cyt c and dATP, and then it oligomerizes into a heptameric complex known as the apoptosome, which recruits and activates cell‐killing caspase‐9/3.^[^ ^22^ ^]^ It has been reported that some endogenous factors, such as calcium, can block the formation of the apoptosome by preventing nucleotide exchange in APAF1.^[^ ^6a^ ^]^ High levels of both nucleotides and mitochondria/cytosol tRNA can interact with Cyt c and inhibit caspase activation,^[^ ^7^ ^]^ impairing cellular responsiveness to pro‐apoptotic stimuli. Here, for the first time, we reported the unexpected finding that a lncRNA, ABL, directly binds the WD1/WD2 domain of APAF1, which competitively blocks the interaction of Cyt c with APAF1 and impairs the formation of the apoptosome, resulting in increased cell survival. Moreover, APAF1 LACE‐seq data further confirmed that APAF1 may function as a newly identified RBP to bind multiple types of RNA including ncRNA. Intriguingly, we found that the APAF1‐binding consense sequence “AACCUU_AG” could be mapped in the 481–540 nt of ABL transcript, which is essential to interact with APAF1. Collectively, these data suggest that multiple types of RNA may be involved in the process of apoptosome assembly to determine cell fate.

Previous studies have reported that upregulation of APAF1 expression improves cisplatin sensitivity, while APAF1 deficiency prevents PTX‐induced apoptosis in cancer.^[^ ^23^ ^]^ It was also revealed that microRNA‐21 transferred from cancer‐associated adipocytes and fibroblasts into ovarian cancer cells suppresses PTX‐induced apoptosis and confers chemoresistance by inhibiting APAF1 expression.^[^ ^24^ ^]^ Here, we found that upregulation of ABL antagonized GC cell apoptosis induced by multiple drugs. Targeting non‐coding RNAs by using a locked nucleic acid (LNA)‐based antisense oligonucleotide strategy has been successfully applied to target miRNAs and lncRNAs in cancer.^[^ ^25^ ^]^ Excitingly, in our study, targeting ABL with nanoencapsulated siRNA could significantly enhance the sensitivity of GC cells to chemotherapy, which indicated the translational value of targeting ABL in a preclinical model.

To date, an increasing number of studies have shown diverse roles for m^6^A modification and its regulators in cancer.^[^ ^17^, ^26^ ^]^ Among these regulators, IGF2BP1/2/3 are the m^6^A readers that target eukaryotic RNAs to maintain RNA stability.^[^ ^18^ ^]^ Our recent study demonstrated that IGF2BP3 recognizes the METTL3‐mediated m^6^A modification on HDGF mRNA and maintains HDGF mRNA stability, which leads to GC cell growth and metastasis to the liver.^[^ ^19^ ^]^ Recently, m^6^A regulators, such as METTL3 and IGF2BPs, have also been reported to execute an m^6^A‐dependent modification of noncoding RNAs involved in cancer development.^[^ ^27^ ^]^ Here, we found that IGF2BP1 directly binds to ABL via the “CACA” motif, which was previously reported as IGF2BP1/3 binding sites.^[^ ^20^ ^]^ Furthermore, IGF2BP1 then recognizes the METTL3‐mediated m^6^A modification on ABL via the “GGAC” motif to enhance ABL stability, which may partly explain the high expression of ABL in GC. Importantly, we also found that combinatorial use of ABL expression and the TNM stage effectively improved the prediction of GC survival, indicating that ABL could be a promising biomarker for GC prognosis.

In summary, our findings reveal an antiapoptotic role for lncRNA ABL in GC, which is associated with GC cell survival and chemotherapeutic drug resistance, resulting in a poor prognosis in patients with GC. Moreover, targeting ABL using nanoencapsulated siRNA combined with chemotherapeutic drugs might represent a novel therapeutic strategy for GC treatment. Therefore, ABL might be a potential predictor and therapeutic target in GC.

## 4 Experimental Section

### Patients and Specimens

All patients in GC cohort 1 or cohort 2 with gastric carcinoma treated with radical gastrectomy without adjuvant radiotherapy or chemotherapy during the observation periods at the Nanjing Drum Tower Hospital, the Affiliated Hospital of Nanjing University Medical School (Nanjing, Jiangsu, China), were included. Cohort 1 included 81 patients who underwent radical gastrectomy between January 3, 2007, and October 2, 2007. The median survival time was 59 months in cohort 1. Cohort 2 included 192 patients who underwent radical gastrectomy between January 14, 2014, and January 4, 2016. The median survival time was 56 months in cohort 2. Paired cancerous GC and normal gastric mucosal tissue specimens were embedded in paraffin to make a tissue microarray (TMA), and clinicopathological features, including age, sex, and TNM stage (American Joint Committee on Cancer [AJCC] classification), were recorded. In addition, 35 fresh‐frozen pathologically confirmed cancerous GC and normal gastric mucosal tissue specimens from recent patients at the Nanjing Drum Tower Hospital were included. These tissues were obtained for qRT‐PCR, RNAscope, RNA‐FISH, and GC organoid culture after written informed consent was provided. This study was approved by the Institutional Review Boards of Nanjing Drum Tower Hospital (20211005).

### Cell Lines and Cell Culture

The AGS and NCI‐N87 GC cell lines were purchased from the American Type Culture Collection (MD, USA), and human HEK293T cells and the SGC7901, BGC823, and MGC803 GC cell lines were obtained from the Type Culture Collection of the Chinese Academy of Sciences (TCCCAS, Shanghai, China). MKN‐45 cells were obtained from the cell bank of the RIKEN BioResource Center (Tsukuba, Japan). SGC7901, NCI‐N87, BGC823, and MKN45 cells were cultured in RPMI‐1640 medium (Invitrogen Life Technologies, CA, USA), MGC803 cells were cultured in DMEM (Invitrogen Life Technologies, CA, USA), and AGS cells were cultured in F12K medium (Cellcook Biotech Co., Ltd., Guangzhou, China). The media for all the cell lines were supplemented with 10% fetal bovine serum (FBS; Gibco, CA, USA), 100 µg mL^−1^ streptomycin, and 100 U mL^−1^ penicillin (New Cell & Molecular Biotech, Suzhou, China), and cells were cultured in an incubator with 5% CO_2_ at 37 °C. Cells were stored at −80 °C using CELLSAVING (New Cell & Molecular Biotech, Suzhou, China). All cells were tested negative for mycoplasma contamination and were authenticated based on STR fingerprinting before use.

### Animal Model

Male BALB/c nude mice (5–6 weeks old) were purchased from Nanjing Biomedical Research Institute of Nanjing University (Nanjing, Jiangsu, China) and maintained in SPF facilities. A total of 2 × 10^6^ BGC823 cells stably overexpressing ABL or lentiviral vectors were subcutaneously injected into the right axilla of nude mice (*n* = 6 per group). Tumor volume was monitored every other day (volume = length × width^2^ × 1/2). Ten days after injection, the mean volume was ≈100 mm^3^, and the mice were treated with or without DDP (4 mg kg^−1^, twice per week). After four treatments, the mice were sacrificed, and the tumors were weighed and imaged. Tumor tissues were then fixed in 4% paraformaldehyde or frozen for further analyses. All animal experiments were performed following a protocol approved by the Institutional Animal Care Committee of Nanjing Drum Tower Hospital.

### Organoid Culture

The organoid culture was first approved by the Institutional Review Board of Nanjing Drum Tower Hospital. The organoid culture method was described previously.^[^ ^28^ ^]^ Briefly, ≈1 cm^3^ GC tissues from three different GC patients were minced, washed with 1× chelating buffer (5.6 × 10^−3^ m Na2HPO4, 8.0 × 10^−3^ m KH2PO4, 96.2 × 10^−3^ m NaCl, 1.6 × 10^−3^ m KCl, 43.4 × 10^−3^ m sucrose, 54.9 × 10^−3^ m d‐sorbitol, and 0.5 × 10^−3^ m d,l‐dithiothreitol (pH = 7)), and cut into 20–50 small pieces. The glands were pressed and then centrifuged for 5 min at 200× *g* and 4 °C. ≈100 glands per 50 µL of basement matrix were seeded in one well of a 24‐well plate warmed to 37 °C. 500 µL of medium containing growth factors (50 ng mL^−1^ EGF, 100 ng mL^−1^ noggin, 1 µg mL^−1^ R‐spondin1, 50% Wnt‐conditioned medium, 200 ng mL^−1^ FGF10, 1 × 10^−9^ m gastrin, 2 × 10^−6^ m TGF‐beta inhibitor, and 10 × 10^−6^ m RHOKi) was carefully added to each well. After organoids formed, they were transfected with ABL overexpression or corresponding control lentiviral vectors for 8 h. Then, the organoids were seeded in 24‐well plates, cultured for one week, and treated with or without DDP (1 µg mL^−1^) for 24 h, and organoid images were acquired with a LEICA DMi8 system. Then, the organoids were fixed, and the expression of ABL, cleaved caspase 3, and Ki67 was detected using the methods described below.

### SiRNA, shRNA, sgRNA, and Plasmid Transfection and Lentiviral transduction

siRNAs targeting ABL, P300, IGF2BP1, IGF2BP3, or METTL3 were designed and synthesized by RiboBio (Guangzhou, China). The sequences are listed in Table S6 (Supporting Information). shRNAs targeting ABL or METTL3 were designed based on siRNA sequences and subcloned into LV3 vectors (pGLV‐h1‐EGFP‐puro), which were constructed by GenePharma (Shanghai, China). ABL‐OE and METTL3‐OE lentiviruses were constructed by GeneChem Co., Ltd. (Shanghai, China) using GV358 vectors (MCS‐3flag‐EGFP‐puro). ABL, APAF1, and IGF2BP1 cDNA constructs as well as corresponding mutants were subcloned into pcDNA3.1 vectors (YouBio, Changsha, China). The ABL plasmid and its corresponding mutants used in RNA pull‐down assays were cloned into a pGEM‐3Z vector using the Xba I/BamH I sites (YouBio, Changsha, China). siRNA was transfected into cells with DharmaFECT4 (Dharmacon, Chicago, USA). All of the plasmids were transfected into cells with Lipofectamine 3000 (Invitrogen, Grand Island, NY). An shRNA‐carrying lentivirus or overexpression lentivirus and their vectors were added to GC cells. 8–12 h later, the lentivirus was removed, and a new culture medium was added. After 72 h, infected GC cells were selected with 1 µg mL^−1^ puromycin (Sigma, USA). To generate BGC823 APAF1 knockout (APAF1‐KO) cells, APAF1 sgRNA (sgRNA sequence: AGCTGCTCTTTGCTGTTGAG) was designed and cloned into a plasmid coexpressing sgRNA and Cas9 (pCas‐puro‐U6‐KO), and the APAF1‐KO cells were created as previously reported.^[^ ^19^ ^]^ Wild‐type METTL3 plasmid was purchased from Addgene (catalog number: 53739, Watertown, MA), and the catalytic mutant (aa395‐398, DPPW→APPA) METTL3 was constructed based on the wild‐type METTL3.

### RNA Sequencing (RNA‐seq)

Total RNA was first extracted from the GC tissues and noncancerous tissues of four GC patients. The quality and quantity of the isolated RNA were assessed by a NanoDropTM ND‐1000. Denaturing agarose gel electrophoresis was used to assess RNA integrity. The total RNA samples were enriched by oligodT (rRNA removal), and then the KAPA Stranded RNA‐Seq Library Prep Kit (Illumina) was used to construct the library. The constructed library was identified by an Agilent 2100 Bioanalyzer and then sequenced by an Illumina HiSeq 4000 sequencer. RNA sequencing was performed by ShuPu Biotechnology LLC (Shanghai, China). The GEO accession number for the high‐throughput sequencing reported in this study is GSE172032.

### LACE‐seq Assay

The detailed experimental steps and analysis of LACE‐seq were described as previously reported.^[^ ^12^ ^]^ The antibodies used are listed in Table S7 (Supporting Information).

### Western Blot and Co‐IP Assay

Western blot and Co‐IP assays were performed as previously reported.^[^ ^29^ ^]^ The antibodies used are listed in Table S7 (Supporting Information).

### ABL‐APAF1 Interaction Assay

2 µg His‐tagged recombinant WD1/WD2 domain of APAF1 was incubated with Ni‐NTA beads at room temperature for 1 h and washed with PBS for three times. Then the beads were incubated with or without ABL sense, anti‐sense, and mutant ABL (∆APAF1) (1 and 2 µg) at 4 °C for 1 h and washed with PBS for three times. Then, 2 µg GST‐tagged recombinant Cyt c was incubated with the beads at room temperature for 1 h and washed with PBS for three times. Beads‐bound proteins and the input were analyzed by Western blot and coomassie blue staining.

### Dot Blot Assay

The dot blot assay was performed as previously reported.^[^ ^19^ ^]^ Methylene blue (MB) was used to interact with mRNA and as the loading control.

### Quantitative Real‐Time RT‐PCR (qRT‐PCR)

Total RNA was extracted from cells or tissues as previously reported^[^ ^29^ ^]^ using TRIzol reagent (Invitrogen, CA, USA). Reverse transcription (RT) was performed with HiScript Q RT SuperMix for qPCR (Vazyme Biotech Co., Ltd., Nanjing, China). RT‐PCR was performed in triplicate with the SYBR Green PCR Kit (Vazyme Biotech Co., Ltd., Nanjing, China) on an Applied Biosystems 7900HT sequence detection system (Applied Biosystems). The primers used are listed in Table S8 (Supporting Information).

### RNA Stability Assay

For the RNA stability assay, actinomycin D (2 µg mL^−1^; MCE, NJ, USA) was used to inhibit transcription. Cells were collected at the indicated time points after treatment with actinomycin D. Then, total RNA was extracted and analyzed by qRT‐PCR. The level of remaining RNA at each time point was normalized to the level measured at the beginning (0 h).

### Proliferation and Drug Sensitivity Assays

Cell proliferation was evaluated by Cell Counting Kit (CCK)‐8 (APExBIO, Houston, USA) and suspension growth assays. In the CCK‐8 assay, 2000 ABL‐overexpressing or ABL‐knockdown cells and corresponding control cells were plated in 96‐well plates. Cell viability was determined at the indicated times according to the manufacturer's instructions for the CCK‐8 assay. For the suspension growth assay, ABL‐overexpressing or ABL‐knockdown and corresponding control GC cells labeled with GFP were seeded in ultralow‐attachment culture plates and incubated for approximately two weeks. Colony numbers were statistically analyzed according to GFP^+^ cells. For the drug sensitivity assay, GC cells were seeded in six‐well plates (500 cells per well), and 12 h later, the cells were treated with cisplatin (DDP), 5‐fluorouracil (5‐Fu), or paclitaxel (PTX) at the indicated doses for 24 h. After incubation at 37 °C for 10–14 d, the cells were fixed with methanol for 30 min and stained with crystal violet (Beyotime, Shanghai, China) for 30 min. DDP was purchased from MedChemExpress (Monmouth Junction, NJ, USA), and 5‐Fu and PTX were purchased from Selleckchem (Houston, USA).

### RNA Fluorescence In Situ Hybridization (RNA‐FISH)

The RNA‐FISH assay was performed as previously described.^[^ ^30^ ^]^ Specific Cy3‐labeled probes for ABL were designed and synthesized for the detection of ABL by RiboBio (Guangzhou, China). After fixation, permeabilization (1× PBS/0.5% Triton X‐100) and prehybridization, cells were hybridized in a hybridization buffer with the Cy3‐labeled probes specific for ABL at 37 °C overnight. The hybridization buffer was then gradually washed off with 4× SSC (including 0.1% Tween‐20), 2× SSC, and 1× SSC at 42 °C. Nuclei were counterstained with 4,6‐diamidino‐2‐phenylindole (DAPI) (Beyotime, Shanghai, China). Confocal images of the cells were captured using Zeiss AIM software and a Zeiss LSM 700 confocal microscope system (Carl Zeiss Jena, Oberkochen, Germany). The expression of ABL in a GC tissue microarray (TMA) was also detected as described above. The proportion of stained cells was categorized into four grades (1, 2, 3, and 4) according to the following criteria: 1, 0%–25% of tumor cells stained with the ABL probe; 2, 25%–50% of tumor cells stained; 3, 50%–75% of tumor cells stained; and 4, 75%–100% tumor cells stained. In addition, the staining intensity was classified into four grades (0, 1, 2, and 3) according to the following standard: 0, all the cells were negative; 1, the stained cells were light red; 2, the stained cells were red; and 3, the stained cells were dark red. The final score for the TMA was calculated as the score for proportion multiplied by the score for intensity. Under these conditions, samples with an IRS of 0–6 or an IRS of 8–12 were classified as having low and high expression of ABL, respectively, in cohorts 1 and 2.

### Immunofluorescence (IF) Staining

The details of the IF assay were described previously.^[^ ^29^ ^]^ Briefly, BGC823 cells were first incubated with an ABL‐specific FISH probe at 37 °C overnight and then with an anti‐APAF1 antibody (1:200, sc‐135836, Santa, USA) or anti‐IGF2BP1 antibody (1:200, 22803‐1‐AP, Proteintech, IL, USA) at 4 °C overnight. Then, the cells were incubated with the corresponding Alexa Fluor‐labeled secondary antibodies (Beyotime, Shanghai, China) at a 1:200 dilution for another 1 h at room temperature. Next, the cells were incubated with DAPI (Beyotime, Shanghai, China) for 5 min. Images of the cells were acquired with a LEICA DMi8 system. To verify whether ABL affects the interaction between APAF1 and Cyt c, BGC823 cells overexpressing ABL and corresponding control cells were treated with DDP (1 µg mL^−1^) for 24 h and then incubated with anti‐APAF1 and anti‐Cyt c antibodies (1:200, ab133504, Abcam, MA, USA) simultaneously. The expression of cleaved caspase‐3 (1:200, #9664, MA, CST, USA) in tissue slides was detected as described above. The antibodies used are listed in Table S7 (Supporting Information).

### RNA Immunoprecipitation (RIP) Assay

The RIP assay was performed as previously described.^[^ ^31^ ^]^ Briefly, the MagnaRIP RNA‐Binding Protein Immunoprecipitation Kit (Millipore, MA, USA) was used according to the manufacturer's instructions. Corresponding cell lysates were incubated with beads coated with 5 µg of control IgG antibody (Beyotime, Shanghai, China), anti‐APAF1 antibody (ab234436, Abcam, MA, USA), or anti‐IGF2BP1 antibody (22803‐1‐AP, Proteintech, IL, USA) with rotation at 4 °C overnight. Next, total RNA was extracted for the detection of ABL expression by qRT‐PCR.

### RNAscope Assay

The RNAscope assay was performed as previously reported.^[^ ^25b^ ^]^ RNAscope probes targeting ABL were designed and purchased from Advanced Cell Diagnostics, and ABL detection was performed according to the manufacturer's instructions for an RNAScope 2.5 HD Definition Assay kit (ACD, USA).

### RNA Pull‐Down and Mass Spectrometry Analysis

The details of the RNA pull‐down assay were described previously.^[^ ^25b^ ^]^ Biotinylated ABL and mutant RNAs were enzymatically digested at the Xba 1 or Bam H1 sites (NEB, USA) and then transcribed in vitro with RNA polymerase (T7 or SP6, Invitrogen, NY, USA) and RNA Biotin Labeling Mix (Roche, Mannheim, Germany). The transcription products were identified by agarose gel electrophoresis and purified by an RNA Clean Concentrator kit (Zymo Research, CA, USA). Streptavidin beads were prepared in accordance with the manufacturer's instructions (Invitrogen, NY, USA). Cell lysates of BGC823 cells treated with anti‐RNase and Protease/Phosphatase Inhibitor Cocktail (Beyotime, Shanghai, China) were incubated with the beads at 4 °C for 4 h, and the RNA‐binding proteins were analyzed using mass spectrometry. RNA pull‐down assays for ABL with recombinant APAF1 (ab198064, Abcam, MA, USA) and IGF2BP1 (H00010642‐P01, Abnova) were also performed as described above.

### In Vitro RNA Pull‐Down Coupled with a Dot Blot Assay

In vitro RNA pull‐down of ABL with a recombinant protein followed by purification of the protein‐bound ABL sequence was performed as previously described.^[^ ^25b^ ^]^ Briefly, in vitro‐transcribed biotinylated ABL was incubated with various recombinant proteins in binding buffer [50 × 10^−3^ m Tris‐HCl (pH 7.9), 10% glycerol, 100 × 10^−3^ m KCl, 5 × 10^−3^ m MgCl_2_, 10 × 10^−3^ m *β*‐ME, and 0.1% NP‐40]. Crosslinking was performed with ultraviolet (UV) light, and then the RNA was partially digested by RNase I (Ambion), allowing a small fragment to remain attached to the protein. RNA‐protein complexes of interest were then partially purified by His‐tag or GST‐tag magnetic beads, and the purified RNA‐protein complexes were treated with proteinase K, which removed the protein but left the RNAs intact. The recovered RNAs were hybridized to Bright Star‐Plus positively charged nylon membranes spotted with 60‐mer antisense DNA oligonucleotides tiling along ABL (sequences listed in Table S8, Supporting Information) at 37 °C overnight. The hybridized membrane was washed and visualized by the detection of streptavidin‐HRP signals.

### Caspase 9 Activity Assay

BGC823 cells overexpressing ABL and corresponding control cells were treated with DDP (1 µg mL^−1^) for 24 h, and then total protein was collected to measure caspase‐9 activity levels using caspase‐9 activity kits (Beyotime, Shanghai, China).

### TUNEL Assay

Apoptosis was assessed using the TUNEL Apoptosis Detection Kit (Beyotime, Shanghai, China) according to the manufacturer's instructions. Cell nuclei were stained with DAPI. Confocal images of cells were sequentially acquired with a LEICA DMi8 system. Cells undergoing apoptosis in tumor tissue slides were also detected as described above.

### Flow Cytometry

GC cells with ABL overexpression or knockdown were treated with DDP at the indicated dose for 24 h, and apoptotic cells were estimated by an Annexin V‐FITC apoptosis kit (Vazyme Biotech Co., Ltd., Nanjing, China) according to the manufacturer's protocol.

### Luciferase Reporter Assay

Cells were seeded in a 24‐well plate at a density of 6 × 10^4^ cells per well before transfection. The cells were cotransfected with a mixture of luciferase reporter vectors (pGL‐SV40‐Rluc‐TK‐Luc) containing ABL‐WT or ABL‐Mut sequences (0.8 µg), IGF2BP1 (0.8 µg), and corresponding control vectors, respectively. After 48 h, the luciferase activity was measured using a dual‐luciferase reporter assay system (Vazyme, Nanjing, China) according to the manufacturer's protocol. The luciferase reporter plasmid was constructed by Cortes Biotechnology (Nanjing, China). ABL‐WT sequence (121‐180 nt region of ABL) is ACTGGAAACGCTACCTACCACGCATTGACTAGG ***A*** CCACAGTGAGGAGGGAACGCTTTAGA; and the ABL‐Mut sequence is ACTGGAAACGCTACCTACCACGCATTGACTAGG ***C*** CCACAGTGAGGAGGGAACGCTTTAGA.

### RNA‐Protein Docking Analysis

The 3D structures of APAF1 and the APAF1‐Cyt c complex were downloaded from the RCSB Protein Data Bank (PDB ID: 3jbt). The catRAPID signature algorithm was utilized to calculate the overall RNA‐binding propensity and RNA‐binding regions for APAF1.^[^ ^32^ ^]^ The secondary structures of the functional fragment of ABL were predicted by the RNAfold and Mfold web servers.^[^ ^33^ ^]^ The 2D structure with the lowest energy score for ABL was used to predict the 3D structure of ABL by 3dRNA software.^[^ ^34^ ^]^ HDOCK is a protein‐RNA docking approach based on a hybrid algorithm of template‐based modeling and ab initio free docking.^[^ ^35^ ^]^ According to the structures of APAF1, IGF2BP1, and ABL, the complex structures of APAF1‐ABL and IGF2BP1‐ABL were predicted by the HDOCK algorithm. Based on energy scores, the complex structure with the lowest energy was selected to analyze interaction sites. The interaction sites in the APAF1‐Cytc and APAF1‐ABL complexes were calculated by Ring software.^[^ ^36^ ^]^

### In Vivo Therapeutic Model

Reagents: 1,2‐Dioleoyl‐3‐trimethylammonium‐propane (DOTAP), 1,2‐dioleoyl ‐sn‐glycero‐3‐phosphoethanolamine (DOPE), and N‐(carbonyl‐methoxy polyethylene glycol 2000)‐1,2‐distearoyl‐sn‐glycerol‐3‐phosphoethanolamine sodium salt (DSPE‐ mPEG2000) were purchased from AVT, cholesterol was purchased from Huixing Biochem, PTX was purchased from Hodo, and protamine was purchased from Sigma‐Aldrich. FAM‐scrRNA was purchased from Biomics Biotech. Preparation of PTX/CL: Paclitaxel‐loaded cationic liposomes (PTX/CLs) were prepared by the thin‐film dispersion method.^[^ ^37^ ^]^ Briefly, DOTAP, DOPE, and cholesterol were dissolved in chloroform at a molar ratio of 2:6:3, and then PTX (4% (w:w) of total lipids) was added. A thin film was formed on the inner wall of the container after the chloroform was removed under reduced pressure. Then, saline was added to hydrate the lipid film to form PTX‐loaded cationic liposomes (PTX/CLs), which were further dispersed under sonication. Cationic liposomes without paclitaxel (CLs) were prepared with the same method, with the PTX step removed from the protocol. The concentration of PTX in PTX/CLs was measured by high‐performance liquid chromatography (HPLC). The chromatographic conditions were as follows: mobile phase: mixture of methanol and water (7:3, v:v); detection wavelength: 227 nm; column temperature: 40 °C; and flow rate: 1 mL min^−1^. Preparation of si‐ABL+PTX/PEG‐CLs: The si‐ABL/protamine complex was prepared by incubating si‐ABL with protamine (N:P = 4, mol:mol) at r.t. for 10 min. si‐ABL+PTX/CLs were prepared by incubating the si‐ABL/protamine complex with PTX/CLs (100 nmol lipids per µg of si‐ABL) at r.t. for 30 min. The si‐ABL+PTX/PEG‐CLs were further modified with DSPE‐mPEG2000 by the postinsertion method.^[^ ^38^ ^]^ si‐ABL+PTX/CLs were incubated with DSPE‐mPEG2000 (5% molar ratio to total lipids) at r.t. for 2 h. Other control CLs (scrRNA+PTX/PEG‐CLs, si‐ABL/PEG‐CLs, and scrRNA/PEG‐CLs) were prepared with the same method, with si‐ABL substituted with scrambled siRNA (scrRNA), PTX/CLs substituted with CLs, or the two components both substituted. The particle size and zeta potential of PTX/CLs and si‐ABL+PTX/PEG‐CLs were measured by a Zetasizer (Malvern Nano ZS90). Their morphologies were examined by a transmission electron microscope (TEM, Hitachi HT7700). Cellular uptake: To determine the cellular uptake of siRNA, BGC823 cells (2 × 10^5^ cells per well) were dispersed and seeded in transparent six‐well plates. After 24 h of culture, the cells were incubated with FAM‐siRNA (100 × 10^−9^ m) or FAM‐siRNA+PTX/PEG‐CLs (with an amount equal to that of FAM‐siRNA) for another 6 h. The cells were lysed with RIPA lysis buffer. The fluorescence intensity of FAM was determined by a microplate reader at 522 nm upon excitation at 494 nm. The concentration of protein was determined by a BCA kit. To determine the cellular uptake of siRNA, cells were incubated with a PTX solution (20 µg mL^−1^ with cremophor EL as the solubilizer) or scrRNA+PTX/PEG‐CLs (with an amount equal to that of the free drugs). The cells were lysed with RIPA lysis buffer, and the concentration of PTX was determined by HPLC. Intracellular delivery: BGC823 cells (2 × 10^5^ cells per well) were dispersed and seeded in confocal dishes. After 24 h of culture, the cells were incubated with FAM‐siRNA/PEG‐CLs (100 × 10^−9^ m). At predetermined time intervals, the cells were washed with PBS, counterstained with LysoTracker Red for 30 min and Hoechst 33342 for 10 min, and then observed with a confocal microscope (Olympus FV3000).

### Statistical Analysis

Statistical analyses were performed with SPSS 18.0 or GraphPad Prism 8.0 software. The differences in IRS for ABL staining in primary tumors and corresponding normal tissues were assessed by the Wilcoxon test (grouped). The probability of differences in OS was ascertained by the Kaplan–Meier method with the log‐rank test for significance. Univariate and multivariate Cox regression analyses were used to estimate hazard ratios (HRs) and the associated 95% confidence intervals (CIs). Then, the predictive value of parameters was analyzed using time‐dependent receiver operating characteristic (ROC) curve analysis for censored data and calculated the AUC of the ROC curves as previously reported.^[^ ^29^ ^]^ Representative data are shown as the mean ± SEM. A two‐tailed unpaired Student's *t*‐test was used for comparison of the difference between the two groups. A one‐way ANOVA test followed by Turkey's multiple comparisons was used for the comparison of differences between more than two groups. *p* < 0.05 was considered statistically significant. *p < 0.05, **p < 0.01, ***p < 0.001, and *p* ≥ 0.05 mean no significance, NS. Experimental reports in the study were reliably reproduced in at least three independent experiments or by multiple biologically independent replicates.

## Conflict of Interest

The authors declare no conflict of interest.

## Authors Contribution

Q.W., C.C., X.X., C.S., and C.C. contributed equally to this work. Q.W., C.C., J.X., A.X., and B.W. performed the experiments; Q.W. and C.C. analyzed data; L.X., K.X., G.X., and X.Z. provided the clinical samples and made TMA; X.X. and R.M. made nanomaterials; Y.X., C.C., and R.S. performed LACE‐seq assay and analyzed the data; C.S. performed RNA/protein–protein docking analysis; Y.F. analyzed the pathological tissues; Z.W. analyzed the TMA data; Q.W., C.C., and and S.W. wrote the paper; B.S., R.M., C.C., Y.X., and W.K. commented on the study and revised the paper; S.W. designed and supervised the research.

## Supporting information

Supporting Information

## Data Availability

The data that support the findings of this study are available from the corresponding author upon reasonable request.
