# Supplementary material for: APAF1‐Binding Long Noncoding RNA Promotes Tumor Growth and Multidrug Resistance in Gastric Cancer by Blocking Apoptosome Assembly
Source: Adv Sci (Weinh). 2022 Aug 17;9(28):2201889. doi: 10.1002/advs.202201889 (PMC9534967; doi:10.1002/advs.202201889)
Supplement: Supplementary file 1 — Supporting Information [file ADVS-9-2201889-s001.pdf]

ADVANCED SCIENCE
Open Access

Supporting Information

for *Adv. Sci.*, DOI 10.1002/advs.202201889

APAF1-Binding Long Noncoding RNA Promotes Tumor Growth and Multidrug Resistance in Gastric Cancer by Blocking Apoptosome Assembly

Qiang Wang, Chen Chen, Xiao Xu, Chuanjun Shu, Changchang Cao, Zhangding Wang, Yao Fu, Lei Xu, Kaiyue Xu, Jiawen Xu, Anliang Xia, Bo Wang, Guifang Xu, Xiaoping Zou, Ruibao Su, Wei Kang, Yuanchao Xue, Ran Mo*, Beicheng Sun* and Shouyu Wang**

Supporting Information

APAF1-binding long non-coding RNA promotes tumor growth and multidrug resistance in gastric cancer by blocking apoptosome assembly

Qiang Wang,[1,2], Chen Chen[3], Xiao Xu[4], Chuanjun Shu[5], Changchang Cao[6], Zhangding Wang[7], Yao Fu[8], Lei Xu[7], Kaiyue Xu[1], Jiawen Xu[3], Anliang Xia[1], Bo Wang[1], Guifang Xu[7], Xiaoping Zou[7], Ruibao Su[6], Wei Kang[9], Yuanchao Xue[6], Ran Mo[4]*, Beicheng Sun[1,2]*, Shouyu Wang[1,3,10]**

This PDF file includes:

Figure S1. ABL expression is elevated in GC.

Figure S2. Identification of the ABL-binding protein.

Figure S3. ABL promotes GC cell survival and multidrug resistance *in vitro*.

Figure S4. Knockdown of ABL promotes GC cell apoptosis by competitively blocking the APAF1 interaction with Cyt c.

Figure S5. IGF2BP1 binds and recognizes the METTL3-mediated m^6A modification on ABL, maintaining ABL stability.

Figure S6. Identification of the effect of ABL-specific siRNA-loaded PEG-CLs.

Figure S7. ABL-specific siRNA-loaded PEG-CLs have no obvious systemic toxicity.

Table S1. Representative up or down-regulated lncRNAs from RNA-seq.

Table S2. Biotinylated ABL sense pull-down followed by MS.

Table S3. Particle size and potential of siRNA/protamine complex.

Table S4. Particle size and potential of siRNA/CLs.

Table S5. Particle size and potential of siRNA/PEG-CLs.

Table S6. The sequences of siRNAs.

Table S7. Antibodies for WB, RIP, IF, IP, and IHC.

Table S8. The oligonucleotides were used in this study.

Supporting Figures

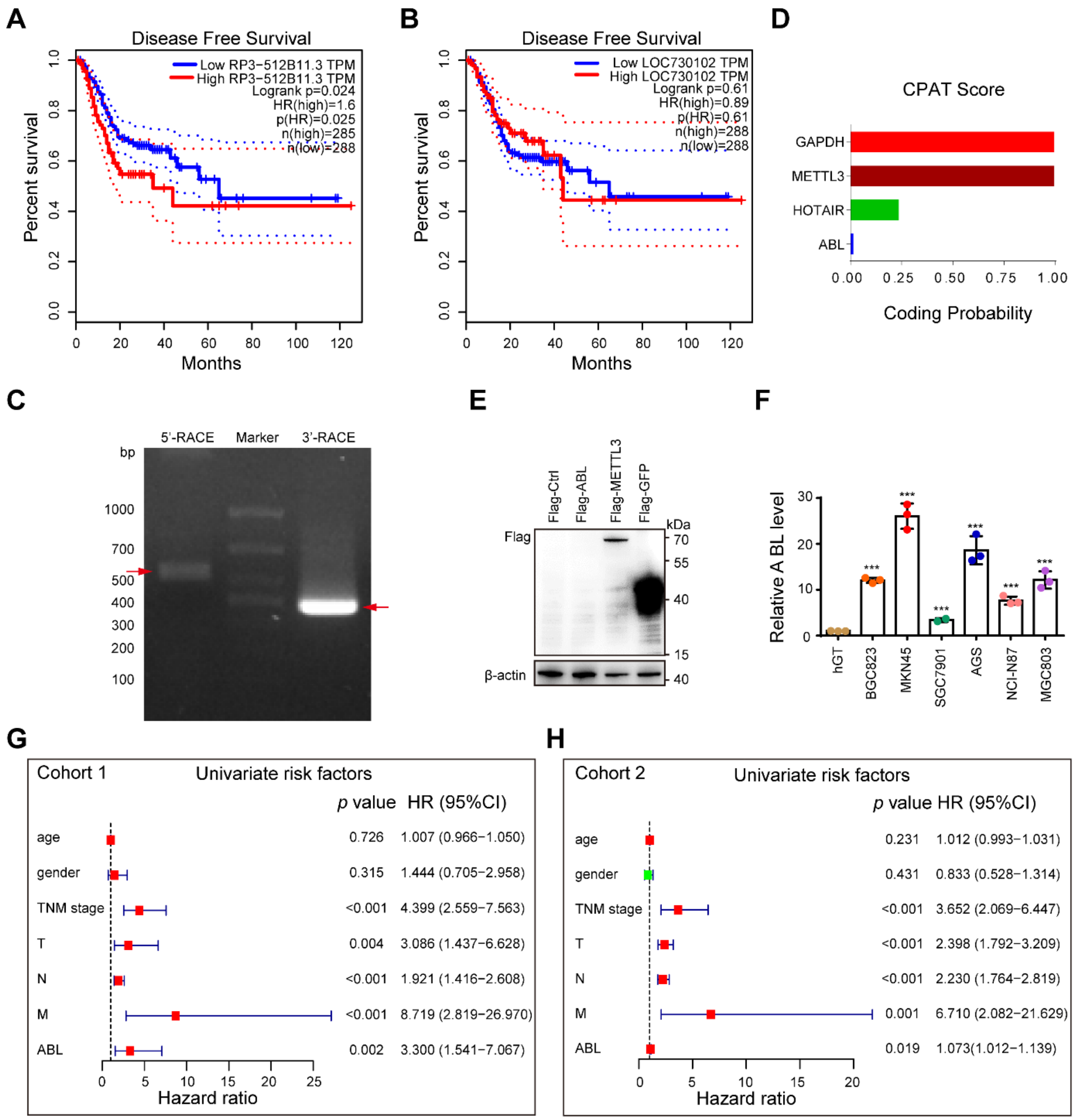

Fig. S1

Figure S1. ABL expression is elevated in GC. A-B) Kaplan-Meier disease-free survival curves based on ABL (A) or LOC730102 (B) expression using the online bioinformatics tool GEPIA (http://gepia.cancer-pku.cn/). C) Representative image of PCR products from the 5'- and 3'-RACE assay. D) The coding capacity of ABL, HOTAIR, METTL3, and GAPDH was determined by the CPAT webserver (http://lilab.research.bcm.edu/cpat/). E) HEK293T cells were transfected with the pcDNA3.1-Flag vector, ABL-Flag (full-length ABL sequence inserted

into the pcDNA3.1 vector), GFP-Flag, or METTL3-Flag. Total cell lysates were resolved in SDS-PAGE gel, and Western blotting for Flag and GAPDH was performed; GFP-Flag and METTL3-Flag were the coding proteins, which were used as positive controls. F) ABL expression was detected in human gastric mucosa tissue (hGT) and different GC cell lines by qRT-PCR. G-H) Univariate analyses were performed for GC cohort 1 (G) and cohort 2 (H). All the bars correspond to 95% confidence intervals (CIs); AUC, area under the curve; CI, confidence interval; GC, gastric cancer; HR, hazard ratio; TNM, tumor, node, metastasis. The probability of differences in DFS was ascertained by the Kaplan-Meier method with the log-rank test (A and B). The data were analyzed by a two-tailed unpaired Student's t-test (F). The data are represented as the means ± SEM. * $p<0.05$; ** $p<0.01$; *** $p<0.001$, NS, no significance.

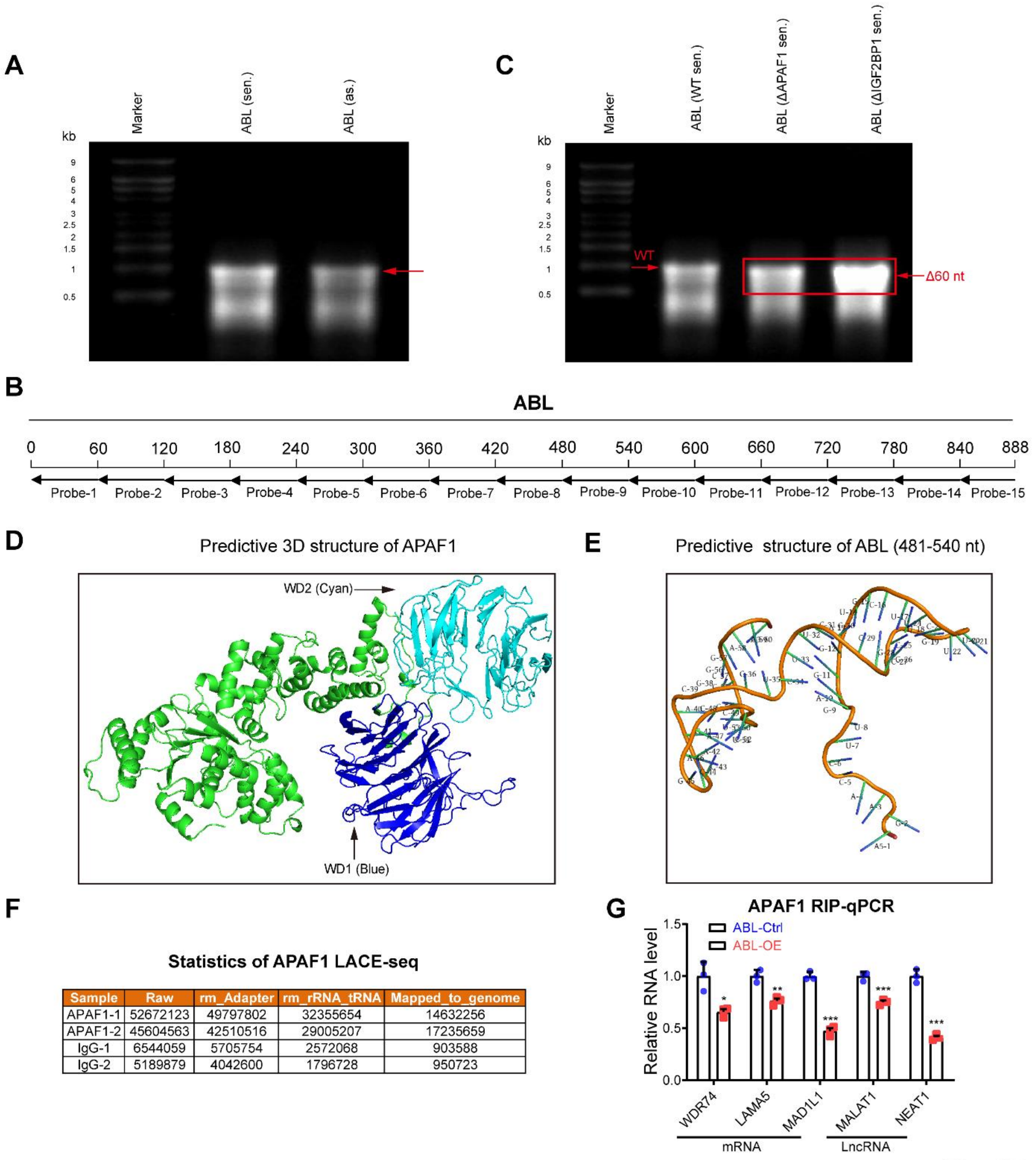

Sample	Raw	rm_Adapter	rm_rRNA_tRNA	Mapped_to_genome
APAF1-1	52672123	49797802	32355654	14632256
APAF1-2	45604563	42510516	29005207	17235659
IgG-1	6544059	5705754	2572068	903588
IgG-2	5189879	4042600	1796728	950723

Fig. S2

Figure S2. Identification of the ABL-binding protein. A) Sense and antisense ABL transcripts were prepared *in vitro* and identified. B) Schematic diagram of ABL probes used in dot blot assay. C) Transcripts for full-length sense ABL, ABL with deletion of nt 481-540, and ABL with deletion of nt 121-160 were prepared *in vitro* and identified. D) Predicted structure of APAF1. E)

Predicted structure of ABL (nt 481-540). F) The number of reads for the APAF1 LACE-seq library. rm_Adapter, after trimming adapters; rm_rRNA_tRNA, after removing rRNA and tRNA-derived reads; Mapped_to_genome, reads mapped to the reference genome. (G) RIP-qPCR analysis was employed to detect the interaction between APAF1 and indicated mRNAs or lncRNAs upon overexpression of ABL. The data were analyzed by a two-tailed unpaired Student's t-test (G). The data are represented as the means ± SEM. * $p<0.05$; ** $p<0.01$; *** $p<0.001$, NS, no significance.

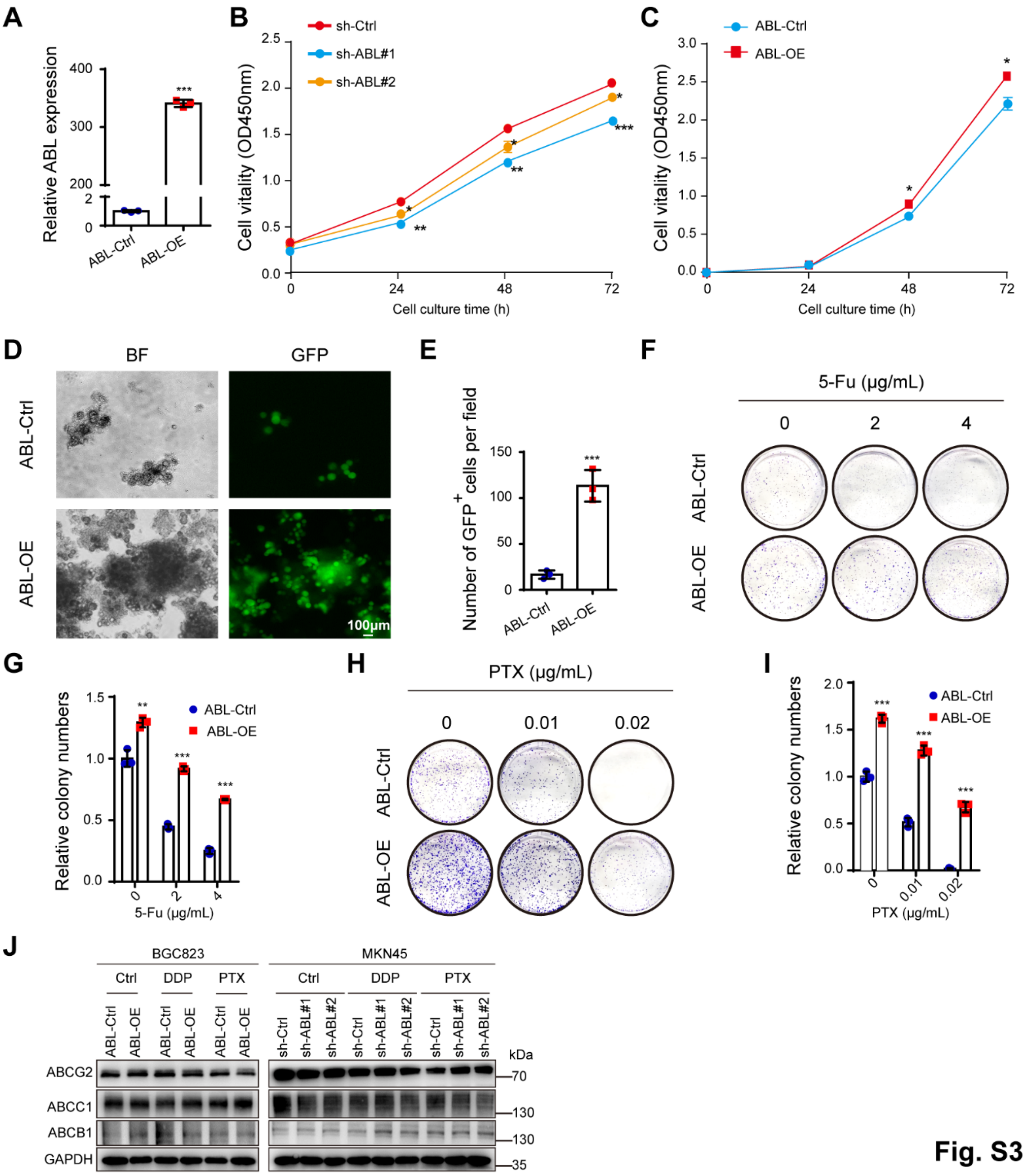

Fig. S3

Figure S3. ABL promotes GC cell survival and multidrug resistance *in vitro*. A) The expression of ABL was examined in ABL-overexpressing BGC823 cells by qRT-PCR. B-C) CCK-8 assays were used to determine the viability of ABL-deficient MKN45 cells (B) and ABL-overexpressing BGC823 cells (C). D) Suspension growth assays were used to determine the cell survival of ABL-overexpressing BGC823 cells (scale bars=100 μm). E) Quantification of the GFP$^+$ cells in (D). F) Overexpression of ABL increased the colony-forming ability of

BGC823 cells and antagonized 5-Fu-induced apoptosis in these cells. G) Quantification of the colony formation assay results in (F). H) Overexpression of ABL increased the colony-forming ability of BGC823 cells and antagonized PTX-induced apoptosis in these cells. I) Quantification of the colony formation assay results in (H). J) Western blotting was applied to determine the expression of ABCB1, ABCC1 and ABCG2 in ABL-overexpressing BGC823 cells or ABL-deficient MKN45 cells after DDP (1 μg/ml) or PTX treatment (0.05 μg/ml) for 24 h. The data were analyzed by a two-tailed unpaired Student's t-test (A-C, E, G, and I). The data are represented as the means ± SEM. * $p<0.05$; ** $p<0.01$; *** $p<0.001$, NS, no significance.

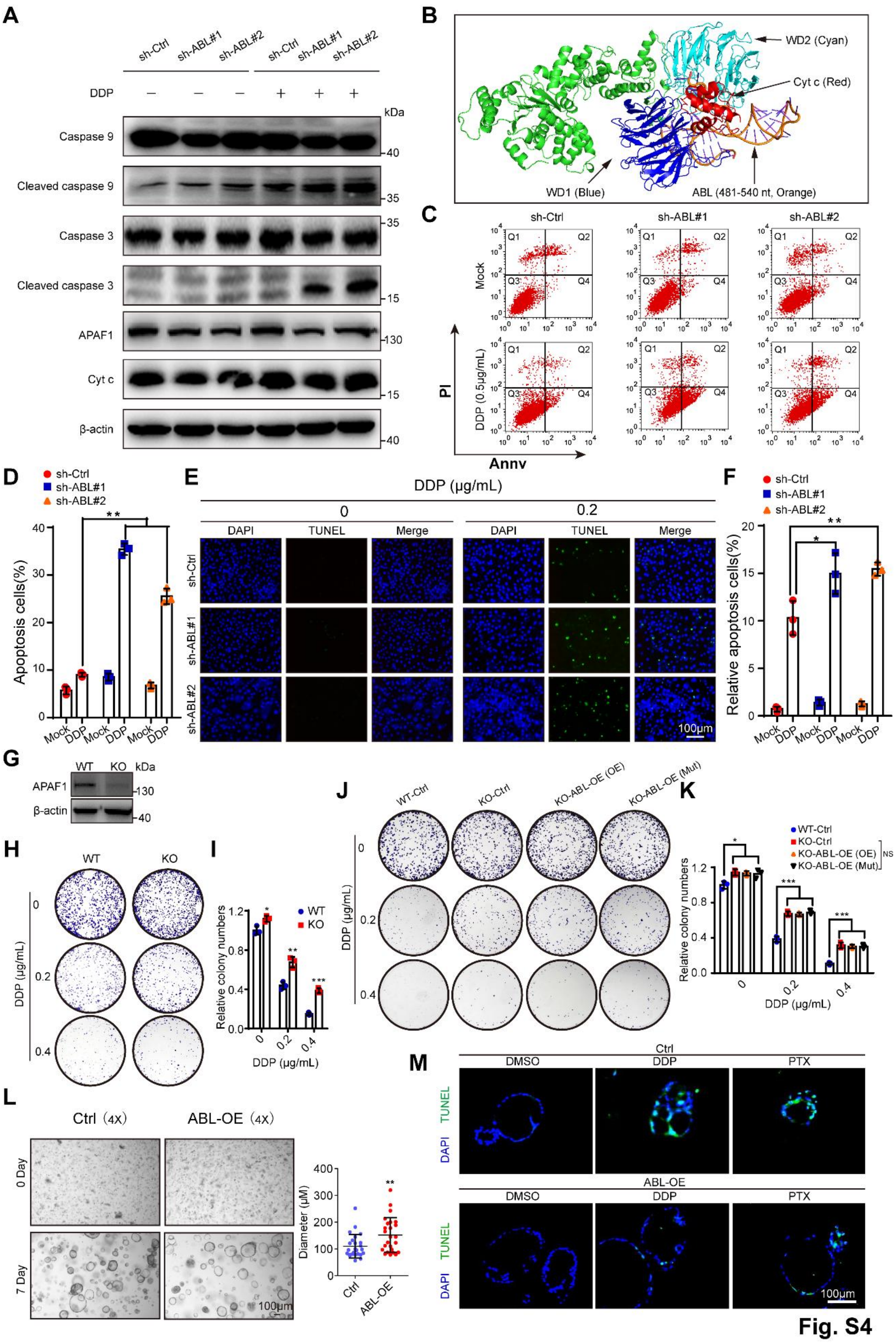

A
sh-Ctrl
sh-ABL#1
sh-ABL#2
DDP
kDa
Caspase 9
Cleaved caspase 9
Caspase 3
Cleaved caspase 3
APAF1
Cyt c
β-actin
B
WD2 (Cyan)
Cyt c (Red)
WD1 (Blue)
ABL (481-540 nt, Orange)
C
Mock
DDP (0.5μg/mL)
PI
Annv
D
Apoptosis cells(%)
E
DDP (μg/mL)
DAPI
TUNEL
Merge
100μm
F
Relative apoptosis cells(%)
G
WT
KO
H
DDP (μg/mL)
I
Relative colony numbers
J
WT-Ctrl
KO-Ctrl
KO-ABL-OE (OE)
KO-ABL-OE (Mut)
K
NS
L
Ctrl (4X)
ABL-OE (4X)
0 Day
7 Day
100μm
Diameter (μM)
M
Ctrl
DMSO
DDP
PTX
ABL-OE

Fig. S4

Figure S4. Knockdown of ABL promotes GC cell apoptosis by competitively blocking the APAF1 interaction with Cyt c. A) Western blotting was applied to determine the expression of the indicated proteins in ABL-deficient MKN45 cells after DDP treatment at 0 or 1 μg/ml for 24 h. B) Docking analysis of protein-protein and RNA-protein interaction models for APAF1 and ABL (nt 481–540) and APAF1-Cyt c. C) An annexin-V-FITC/PI assay was used to detect apoptotic ABL-deficient MKN45 cells after DDP treatment at 0 or 1 μg/ml for 24 h. D) Quantification of the apoptotic cells in (C). E) A TUNEL assay was used to detect apoptotic ABL-deficient MKN45 cells after DDP treatment at 0 or 1 μg/ml for 24 h. F) Quantification of the apoptotic cells in (E). G) The protein levels of APAF1 in BGC823 cells with APAF1 knockout were measured by western blotting. H) Knockout of APAF1 increased the colony-forming ability of BGC823 cells and antagonized DDP-induced apoptosis in these cells. I) Quantification of the colony formation assay results in (H). J) The colony-forming ability was detected in APAF1 knockout BGC823 cells transfected with ABL-overexpressing WT, Mut plasmids, or their corresponding controls after DDP treatment at 0, 0.2, 0.4 μg/ml for 24 h. K) Quantification of the colony formation assay results in (J). L) Representative images of GC organoids transfected with ABL overexpression vectors or a control lentivirus at 0 or 7 days (scale bars=100 μm, left panel, n=3) and the quantification of organoid diameters (right panel). M) Sections of organoids from different groups underwent TUNEL staining (scale bars=100 μm). The data were analyzed by a two-tailed unpaired Student’s t-test (D, F, I, K, and L). The data are represented as the means ± SEM. * $p<0.05$; ** $p<0.01$; *** $p<0.001$, NS, no significance.

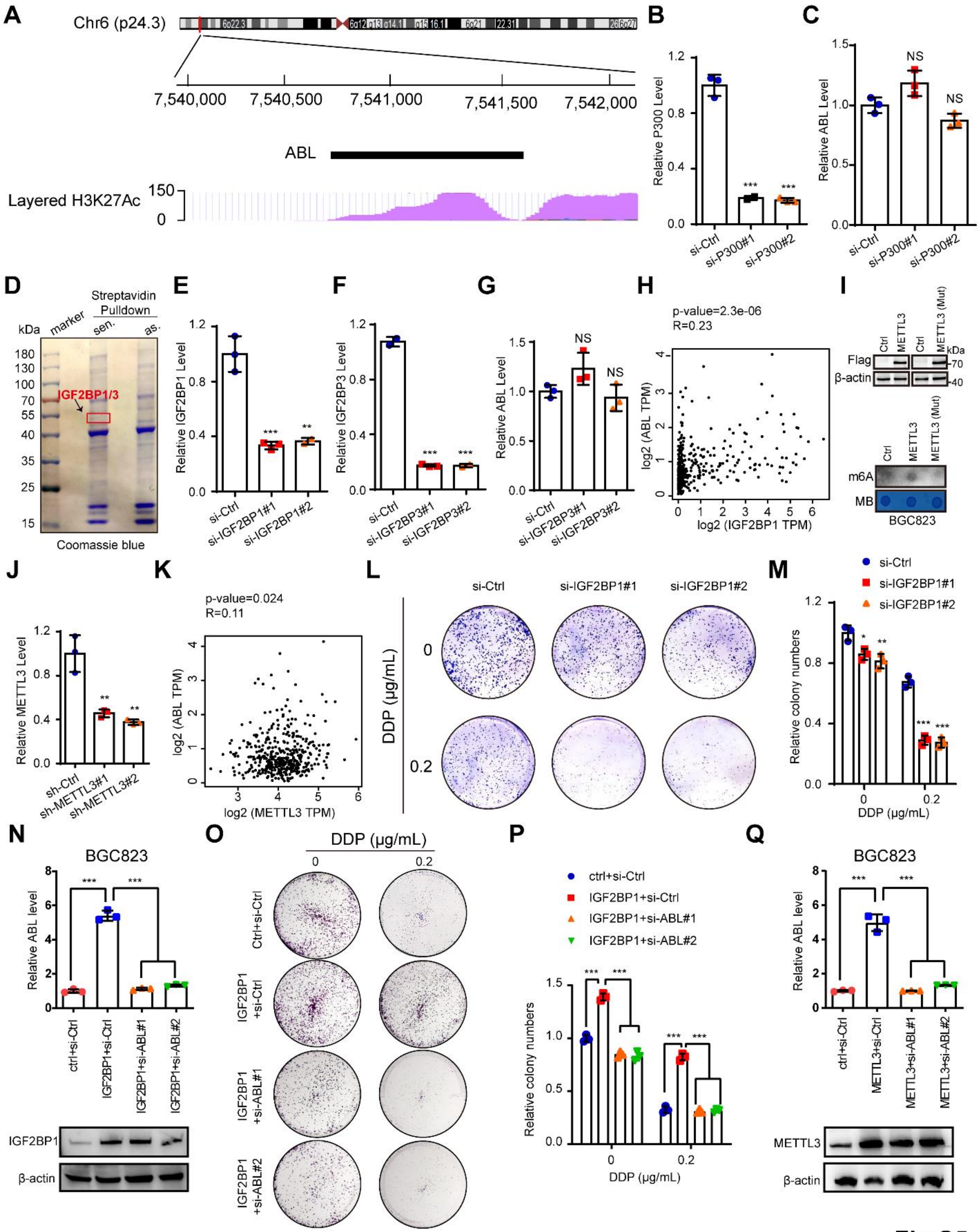

Fig.S5

Figure S5. IGF2BP1 binds and recognizes the METTL3-mediated m6A modification on ABL, maintaining ABL stability. A) Data from the UCSC genome bioinformatics site

(http://genome.ucsc.edu/) showed enrichment of H3K27ac in the promoter of ABL. B) P300 knockdown efficiency was verified in BGC823 cells by qRT-PCR. C) The ABL levels in BGC823 cells with P300 deficiency were determined by qRT-PCR. D) Coomassie brilliant blue staining of proteins pulled down by biotinylated ABL. E-F) IGF2BP1 and IGF2BP3 knockdown efficiencies in BGC823 cells were verified by qRT-PCR. G) The ABL levels in BGC823 cells with IGF2BP3 deficiency were determined by qRT-PCR. H) IGF2BP1 expression was positively correlated with ABL expression in GC (linear regression) using the online bioinformatics tool GEPIA (http://gepia.cancer-pku.cn/). TPM, transcripts per million. I) The protein levels of Flag-METTL3 in BGC823 cells with wild-type or catalytic mutant (Mut) METTL3 overexpression were measured by western blotting (upper panel). The mRNAs isolated from wild-type or catalytic mutant METTL3-overexpressing GC cells were used in dot blot analyses with an m^6A antibody (bottom panel). MB (methylene blue) staining served as a loading control. J) The knockdown efficiencies of METTL3 were verified in MKN45 GC cells by qRT-PCR. K) METTL3 expression was positively correlated with ABL expression in GC (linear regression) using the online bioinformatics tool GEPIA (http://gepia.cancer-pku.cn/). L) Knockdown of ABL decreased the colony-forming ability of BGC823 cells and promoted DDP-induced apoptosis in these cells. M) Quantification of the colony formation assay results in (L). N) The knockdown efficiency of ABL using specific siRNAs in IGF2BP1-overexpressing BGC823 cells was detected by qRT-PCR (upper panel); The protein levels of IGF2BP1 in BGC823 cells with IGF2BP1 overexpression were measured by western blotting (bottom panel). O) Representative images of the cell colony formation abilities of IGF2BP1-overexpressing BGC823 cells transfected with ABL-specific siRNAs or corresponding controls and then treated with DDP at the indicated doses for 24 h. P) Quantification of the colony formation assay results in (O). Q) The knockdown efficiency of ABL using specific siRNAs in METTL3-overexpressing BGC823 cells was detected by qRT-PCR (upper panel); The protein levels of METTL3 in BGC823 cells with METTL3 overexpression were measured by western blotting (bottom panel). The data were analyzed by a two-tailed unpaired Student’s t-test (B, C, E-G, J, M, N, P, and Q). The data are represented as the means ± SEM. * $p<0.05$; ** $p<0.01$; *** $p<0.001$, NS, no significance.

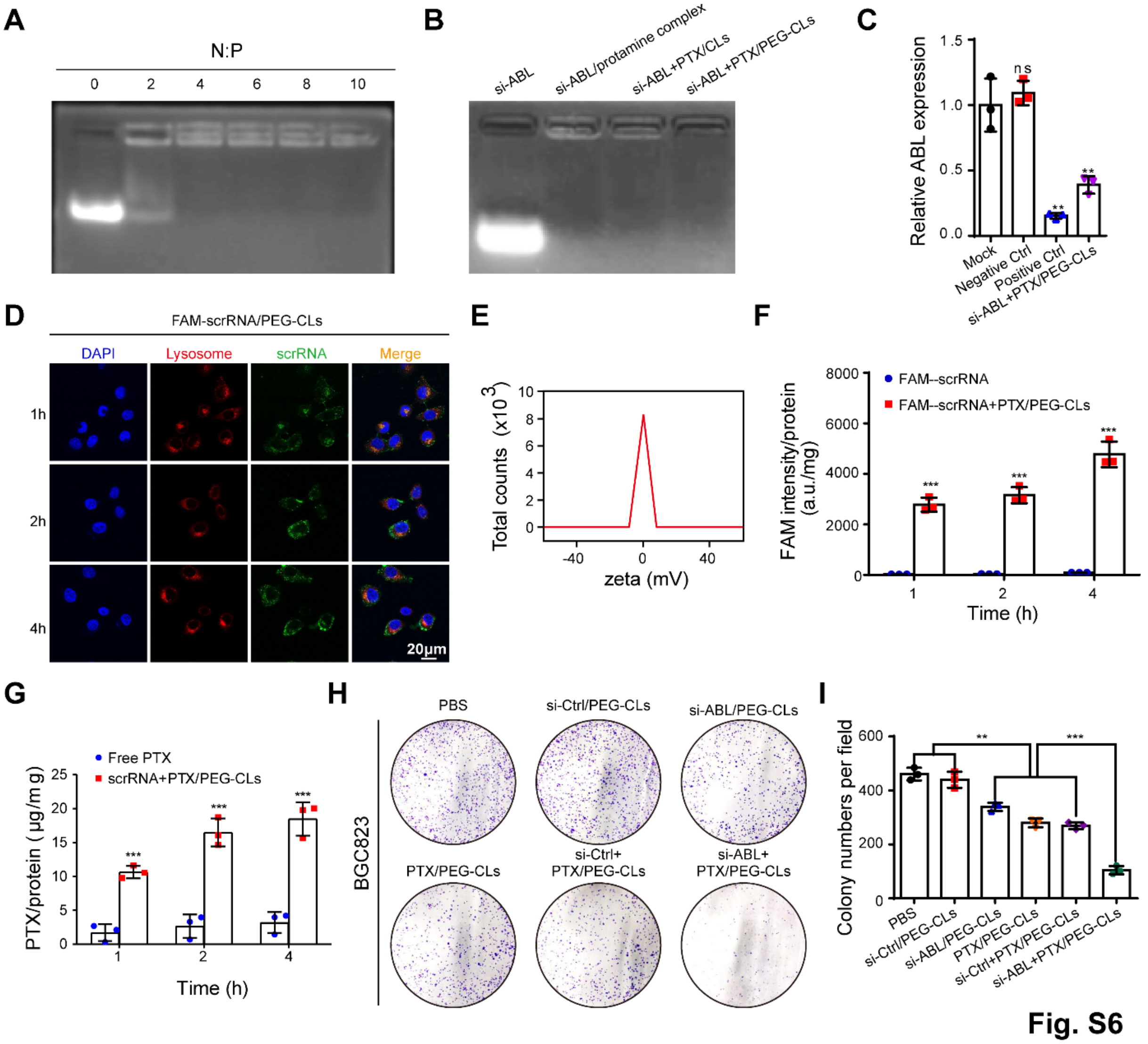

Figure S6. Identification of the effect of ABL-specific siRNA-loaded PEG-CLs. A) Agarose gel electrophoresis of the siRNA/protamine complex at different N:P ratios. B) Agarose gel electrophoresis of si-ABL, si-ABL/protamine complex, si-ABL+PTX/CLs, and si-ABL+PTX/PEG-CLs. C) The postinsertion group showed no obvious effect on transfection efficiency, indicating that the postinsertion method is better for preparing PEG-CLs. D) Intracellular delivery of siRNA PEG-CLs or free siRNA was detected at the indicated times by IF (scale bars=20 μm). E) Zeta potential of ABL-specific siRNA-loaded PEG-CLs. F-G) The cellular uptake of free siRNA, PTX, and siRNA+PTX/PEG-CLs was detected. H) A colony formation assay was used to assess the *in vitro* antitumor capability of si-ABL+PTX/PEG-CLs. I) Quantification of the colony formation assay results in (H). The data were analyzed by a

two-tailed unpaired Student's t-test (C, F, G, and I). The data are represented as the means ± SEM. * p<0.05; ** p<0.01; *** p<0.001, NS, no significance.

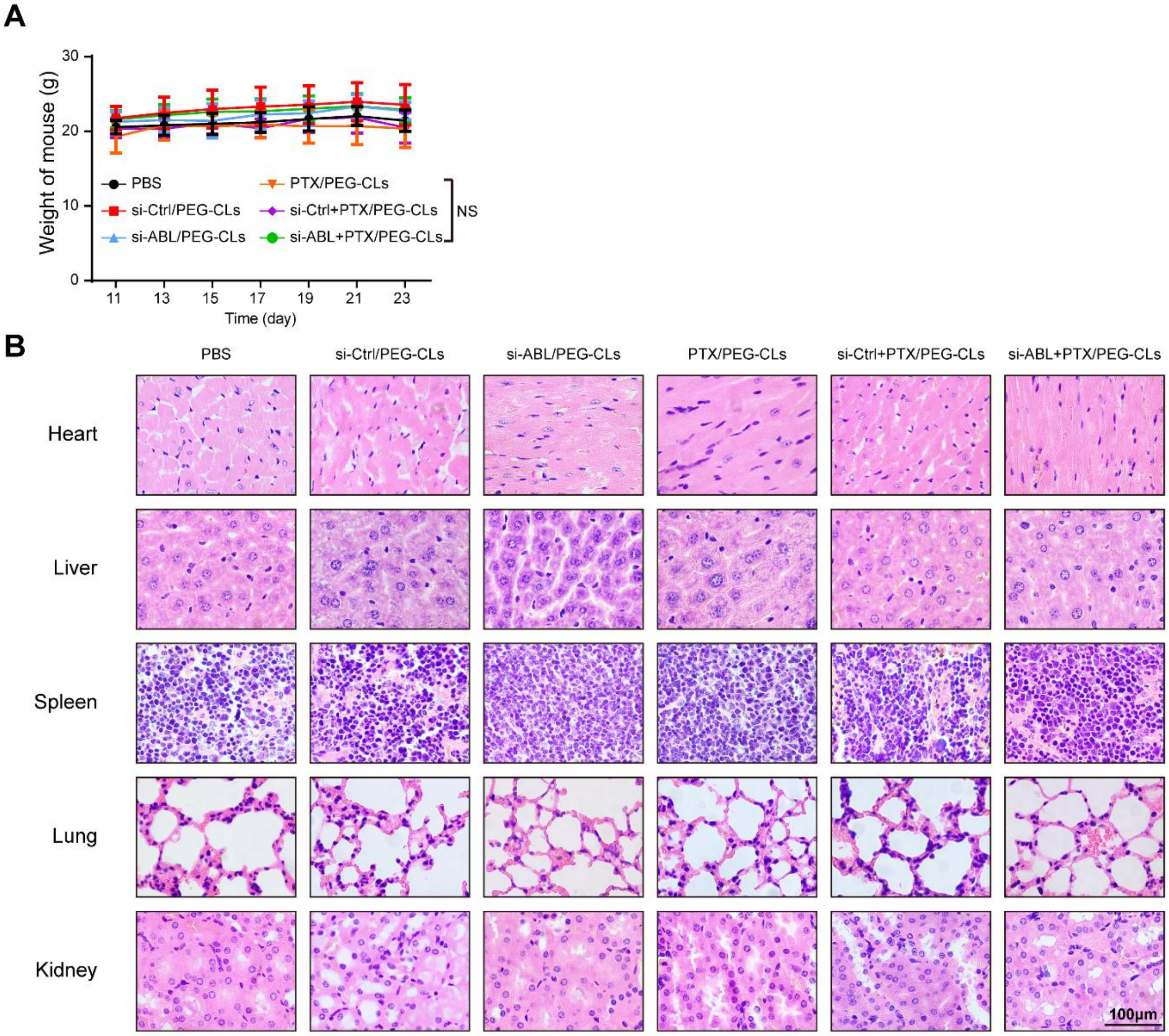

Fig. S7

Figure S7. ABL-specific siRNA-loaded PEG-CLs have no obvious systemic toxicity. A) Mouse weight was monitored every other day in each group (n=6). B) Representative H&E staining of major organs, including the heart, liver, spleen, lungs, and kidneys, from mice in each group at the end of the experiment. The data were analyzed by one-way ANOVA test followed by Turkey's multiple comparisons (A). The data are represented as the means ± SEM. * p<0.05; ** p<0.01; *** p<0.001, NS, no significance.

Supporting Tables

Table S1. Representative up or down-regulated lncRNAs from RNA-seq

**LncRNA Annotation**		**Differential Expression**			
**GeneSymbol**	**source**	**log2FC**	**Fold_Change**	**p_value**	**q_value**
RP3-512B11.3	GENCODE	2.348886063	5.094307562	1.40887E-05	0.039354458
LOC730102	RefSeq	1.990616039	3.974066569	1.8763E-05	0.043010915
RP11-37B2.1	GENCODE	1.305542559	2.471766665	0.001497517	0.158735494
LOC100288637	RefSeq	1.162140427	2.23789202	0.001645347	0.162808241
RP11-1275H24.3	GENCODE	1.167614434	2.246399367	0.001821883	0.16566429
DUXAP10	RefSeq	2.675223575	6.387376868	0.002724512	0.185397161
LINC00543	GENCODE	1.282344685	2.432339625	0.004594469	0.203758381
RP11-803D5.4	GENCODE	1.358719515	2.56457456	0.004865512	0.207919361
RP11-649A18.4	GENCODE	1.570332954	2.969732436	0.008310237	0.236849722
LINC01296	RefSeq	1.725211187	3.306285223	0.01347973	0.267398003
MIR4435-2HG	RefSeq	1.038804638	2.054524648	0.015748694	0.277687696
RP11-589N15.2	GENCODE	1.217501639	2.325436652	0.016904124	0.281968239
CTA-384D8.34	GENCODE	1.17969342	2.265286334	0.017907056	0.286048666
LOC401585	RefSeq	2.345952443	5.083959168	0.019422357	0.291005

					463
LINC01572	RefSeq	1.048726356	2.068702744	0.026202525	0.310253643
RP11-649A18.5	GENCODE	1.081856016	2.116757523	0.027562718	0.313953485
ABHD11-AS1	RefSeq	1.83811065	3.57541486	0.02943696	0.317886269
LINC00152	RefSeq	1.366649814	2.578710494	0.030982672	0.320545784
RP11-79H23.3	GENCODE	1.324402887	2.504292168	0.033006479	0.324641191
LL22NC03-N14H11.1	GENCODE	1.672258134	3.187130592	0.036003641	0.332974077
RP11-797A18.6	GENCODE	1.428063306	2.69085249	0.037935589	0.337272614
AC017002.2	GENCODE	1.281113686	2.430265083	0.038046265	0.337272614
MIR4435-2HG	RefSeq	1.232520427	2.349771429	0.040180489	0.342033401
GSE61474_XLOC_058624	RNA-seq: Clark et al 2015	1.210444469	2.314089188	0.040458719	0.343015034
LINC00857	RefSeq	1.058650108	2.082981619	0.04302053	0.348952716
RP11-37B2.1	GENCODE	1.492443998	2.813652186	0.043948088	0.350914696
RP11-626H12.1	GENCODE	2.317480806	4.984610605	0.0443468	0.352218925
LOC400043	RefSeq	-2.21287222	0.21570444	0.043595338	0.349999719
PWAR5	RefSeq	-2.03395636	0.244184518	0.024637721	0.308751

					826
PWAR6	GENCODE	-1.76530452	0.294164586	0.019661114	0.291746778
GSE61474_XLOC_020344	RNA-seq: Clark et al 2015	-1.734616	0.300488983	0.030932554	0.320374888
PART1	RefSeq	-1.70357706	0.307023914	0.006058087	0.2213024
MIR4458HG	RefSeq	-1.07645654	0.474192075	0.025287889	0.309135684
LINC01184	RefSeq	-1.04697879	0.483980628	0.032289326	0.323317663

Table S2. Biotinylated ABL sense pull-down followed by MS

**Band 1 for peptides identification**										
**Accession**	**Description**	**Score**	**Coverage**	**Proteins**	**Unique Peptides**	**Peptides**	**PSMs**	**AAs**	**MW [kDa]**	**calc. pI**
O14727-3	Isoform 3 of Apoptotic protease-activating factor 1 OS=Homo sapiens OX=9606 GN=APAF1 - [APAF_HUMAN]	613.46	65.49	8	70	70	216	1194	135.9	6.44
Q02413	Desmoglein-1 OS=Homo sapiens OX=9606 GN=DSG1 PE=1 SV=2 - [DSG1_HUMAN]	2.83	1.53	1	1	1	1	1049	113.7	5.03
Q14	Desmoglein-2 OS=Homo sapiens OX=9606	2.	5.	1	4	4	5	1	12	5.

126	GN=DSG2 PE=1 SV=2 - [DSG2_HUMAN]	84	19					1118	2.2	24
P42166	Lamina-associated polypeptide 2, isoform alpha OS=Homo sapiens OX=9606 GN=TMPO PE=1 SV=2 - [LAP2A_HUMAN]	1.70	1.59	1	1	1	1	694	75.4	7.66
P08238	Heat shock protein HSP 90-beta OS=Homo sapiens OX=9606 GN=HSP90AB1 PE=1 SV=4 - [HS90B_HUMAN]	1.69	4.01	7	2	2	2	724	83.2	5.03
Q9Y2W1	Thyroid hormone receptor-associated protein 3 OS=Homo sapiens OX=9606 GN=THRAP3 PE=1 SV=2 - [TR150_HUMAN]	1.63	1.15	1	1	1	1	955	108.6	10.15
P02545-5	Isoform 5 of Prelamin-A/C OS=Homo sapiens OX=9606 GN=LMNA - [LMNA_HUMAN]	1.61	5.31	9	2	2	2	565	62.8	6.18
Q13045-2	Isoform 2 of Protein flightless-1 homolog OS=Homo sapiens OX=9606 GN=FLII - [FLII_HUMAN]	1.61	0.74	3	1	1	1	1214	138.4	6.07
P78347-2	Isoform 2 of General transcription factor II-I OS=Homo sapiens OX=9606 GN=GTF2I - [GTF2I_HUMAN]	0.00	0.94	4	1	1	1	957	107.9	7.94
P27987	Inositol-trisphosphate 3-kinase B OS=Homo sapiens OX=9606 GN=ITPKB PE=1 SV=5 - [IP3KB_HUMAN]	0.00	2.22	1	1	1	3	946	102.3	8.43
Q96L73-2	Isoform 2 of Histone-lysine N-methyltransferase, H3 lysine-36 and H4 lysine-20 specific OS=Homo sapiens OX=9606 GN=NSD1 - [NSD1_HUMAN]	0.00	0.58	3	1	1	1	2427	267.2	8.51

**Band 2 for peptides identification**										
Q9NZI8	Insulin-like growth factor 2 mRNA-binding protein 1 OS=Homo sapiens OX=9606 GN=IGF2BP1 PE=1 SV=2 - [IF2B1_HUMAN]	14.15	7.80	9	2	3	7	577	63.4	9.20
A0A087WWT3	Serum albumin OS=Homo sapiens OX=9606 GN=ALB PE=1 SV=1 - [A0A087WWT3_HUMAN]	4.40	3.79	9	1	1	1	396	45.1	6.10
F5GWG3	Retinoic acid-induced protein 3 (Fragment) OS=Homo sapiens OX=9606 GN=GPRC5A PE=1 SV=8 - [F5GWG3_HUMAN]	2.41	4.76	2	1	1	1	273	30.5	9.03
Q07666-3	Isoform 3 of KH domain-containing, RNA-binding, signal transduction-associated protein 1 OS=Homo sapiens OX=9606 GN=KHDRBS1 - [KHDR1_HUMAN]	2.27	3.47	3	1	1	1	404	44.0	7.28
H0YJH9	Polyadenylate-binding protein 2 (Fragment) OS=Homo sapiens OX=9606 GN=PABPN1 PE=1 SV=1 - [H0YJH9_HUMAN]	2.22	11.46	6	1	1	1	96	11.1	11.27
P50990-3	Isoform 3 of T-complex protein 1 subunit theta OS=Homo sapiens OX=9606 GN=CCT8 - [TCPQ_HUMAN]	2.18	2.11	3	1	1	1	475	51.6	5.24
B4DNK4	Pyruvate kinase OS=Homo sapiens OX=9606 GN=PKM PE=1 SV=1 - [B4DNK4_HUMAN]	2.14	23.19	9	7	7	7	457	49.9	7.83
Q8IVT2	Mitotic interactor and substrate of PLK1 OS=Homo sapiens OX=9606 GN=MISP PE=1 SV=1 - [MISP_HUMAN]	2.07	2.36	1	1	1	1	679	75.3	6.83
P06	Isoform 3 of Tyrosine-protein kinase Fyn	2.	2.	7	1	1	2	4	54.	6.

241-3	OS=Homo sapiens OX=9606 GN=FYN - [FYN_HUMAN]	04	28					82	5	23
Q15646	2'-5'-oligoadenylate synthase-like protein OS=Homo sapiens OX=9606 GN=OASL PE=1 SV=2 - [OASL_HUMAN]	2.03	6.03	3	2	2	2	514	59.2	7.87
D6R9W4	Drebrin (Fragment) OS=Homo sapiens OX=9606 GN=DBN1 PE=1 SV=1 - [D6R9W4_HUMAN]	2.01	6.62	5	1	1	1	317	36.4	5.14
P38646	Stress-70 protein, mitochondrial OS=Homo sapiens OX=9606 GN=HSPA9 PE=1 SV=2 - [GRP75_HUMAN]	1.95	1.62	1	1	1	1	679	73.6	6.16
A0A140T9R1	Flotillin-1 (Fragment) OS=Homo sapiens OX=9606 GN=FLOT1 PE=1 SV=1 - [A0A140T9R1_HUMAN]	1.87	12.32	17	3	3	3	357	39.8	6.37
Q8WWL7	G2/mitotic-specific cyclin-B3 OS=Homo sapiens OX=9606 GN=CCNB3 PE=1 SV=2 - [CCNB3_HUMAN]	1.70	0.93	1	1	1	1	1395	157.8	6.68
P61158	Actin-related protein 3 OS=Homo sapiens OX=9606 GN=ACTR3 PE=1 SV=3 - [ARP3_HUMAN]	1.63	6.22	4	2	2	2	418	47.3	5.88
Q9NUL7	Probable ATP-dependent RNA helicase DDX28 OS=Homo sapiens OX=9606 GN=DDX28 PE=1 SV=2 - [DDX28_HUMAN]	0.00	5.19	1	1	1	1	540	59.5	10.42
P35659-2	Isoform 2 of Protein DEK OS=Homo sapiens OX=9606 GN=DEK - [DEK_HUMAN]	0.00	7.33	6	2	2	2	341	38.7	8.15
P06733-	Isoform MBP-1 of Alpha-enolase OS=Homo sapiens OX=9606 GN=ENO1 -	0.0	3.52	2	1	1	1	34	36.9	6.2

2	[ENOA_HUMAN]	0	1	8

Table S3. Particle size and potential of siRNA/protamine complex

**N:P (mol:mol)**	**2**	**4**	**6**	**8**	**10**
Size (nm)	267.9±8.4	246.4±6.1	226.1±22.9	187.5±13.7	167.4±7.8
Zeta (mV)	-27.0±2.1	-18.6±0.6	-1.7±1.2	9.7±2.3	16.8±4.8

Table S4. Particle size and potential of siRNA/CLs

**Lipid (nmol/μg siRNA)**	**50**	**100**
Size (nm)	146.5±5.8	68.5±1.7
Zeta (mV)	27.2±10.7	34.3±3.1

Table S5. Particle size and potential of siRNA/PEG-CLs

$\mathbf{n_{PEG}/n_{total\ lipids}}$	**1%**	**3%**	**5%**
Size (nm)	68.9±2.1	71.0±1.0	73.1±1.9
Zeta (mV)	27.7±4.6	10.0±0.3	0.17±0.61

Table S6. The sequences of siRNAs

siRNAs	Sequences (5'-3')
ABLsiRNA#1	GCGAAGAACCCTAGGCAGA
ABLsiRNA#2	ACCCTCTCCGGAACTCAGA
P300 siRNA#1	CGACTTACCAGATGAATTA
P300 siRNA#2	GCACAAATGTCTAGTTCTT
METTL3 siRNA#1	CGACTACAGTAGCTGCCTT
METTL3 siRNA#2	CTGCAAGTATGTTCACTATGA
IGF2BP1 siRNA#1	GGCTCAGTATGGTACAGTA
IGF2BP1 siRNA#2	TGAAGATCCTGGCCCATAA
IGF2BP3 siRNA#1	GCTGAGAAGTCGATTACTA
IGF2BP3 siRNA#2	TAAGGAAGCTCAAGATATA

Table S7. Antibodies for WB, RIP, IF, IP, and IHC

**Antibodies**	**Source**	**Identifier**
anti-GAPDH (for WB)	Beyotime	Cat#AG019
anti-APAF1 (for WB, RIP and LACE)	Abcam	Cat#ab234436
anti-APAF1 (for IF and IP)	Santa	Cat#sc-135836
anti-IGF2BP1 (for WB, RIP and IF)	Proteintech	Cat#22803-1-AP
anti-METTL3 (for WB)	Proteintech	Cat# 15073-1-AP
anti-Rabbit IgG (for RIP and LACE)	Beyotime	Cat#A7016
anti-Mouse IgG (for RIP)	Beyotime	Cat#A7028
anti-HRP mouse (for WB)	Beyotime	Cat#A0216
anti-HRP rabbit (for WB)	Beyotime	Cat#A0208
anti-IHC rabbit (for IHC)	Servicebio	Cat#G1215
anti-β-actin (for WB)	Beyotime	Cat#AA128
anti-caspase-9 (for WB)	CST	Cat#9509
anti-caspase-3 (for WB)	CST	Cat#9662
anti-cleaved caspase-3 (for IF)	CST	Cat#9664
anti-Cyt c (for WB and IF)	Abcam	Cat#ab133504
anti-Ki-67 (for IHC)	Servicebio	Cat#GB111499
anti-HA (for WB)	Beyotime	Cat#AF5057
anti-His (for WB)	Beyotime	Cat#P2233
anti-FLAG (for WB)	Sigma -Aldrich	Cat#F1804
anti-m6A (for RIP and Dot blot)	Abcam	Cat#ab208577
anti-GST (for WB)	Abmart	Cat#MY1901
anti-His (for WB)	SMART	Cat# SLAB28

Table S8. The oligonucleotides were used in this study

**Names**	**Sequences (5'-3')**
**qPCR primers for gene expression**	
ABL F[a]	TGAGGATGCTTGTCTCTCGC
ABL R[b]	GAATGTTCAAGCTCCGCGTC
GAPDH F	CATGTGGGCCATGAGGTCCACCAC
GAPDH R	GGGAAGCTCACTGGCATGGCCTTCC
METTL3 F	ATCCCCAAGGCTTCAACCAG
METTL3 R	AGGGTGATCCAGTTGGGTTG
P300 F	GCAGTGTGCCAAACCAGATG
P300 R	CATAGCCCATAGGCGGGTTG
IGF2BP1 F	AGCTCCTTTATGCAGGCTCC
IGF2BP1 R	CCGGGAGAGCTGTTTGATGT
IGF2BP3 F	ACTGCACGGGAAACCCATAG
IGF2BP3 R	CCAGCACCTCCCACTGTAAAT
WDR74 F	CCTGGGGTGTGTAGGATGC
WDR74 R	CAAGTCCAGCCAGTCATTCCG
LAMA5 F	GACTGCCAACAGTGCCAAC
LAMA5 R	CCACCCTGATAGGTGCCAT
MAD1L1 F	TGGACTGGATATTTCTACCTCGG
MAD1L1 R	CCTCACGCTCGTAGTTCCTG
MALAT1 F	AAAGCAAGGTCTCCCCACAAG
MALAT1 R	GGTCTGTGCTAGATCAAAAGGCA
NEAT1 F	CCAGTTTTCCGAGAACCAAA
NEAT1 R	ATGCTGATCTGCTGCGTATG
SCARNA2 F	GATCTTATTTGATCGGATCGTG
SCARNA2 R	CAATTCATCACTTCTGAGCGC
**ABL probes used in *in vitro* RNA pull-down coupled with dot-blot assay**	
Probe-1	CTATTTCCGCCCGGGGACCGAGGAAATCCTCAAAAGCCG GGCCACGCATTCCCAGCCCGA

	GATCAACAAAAGTAAATATTTGACCAGTAAGTCTCCAAT
Probe-2	CGAGAGGCCCCTCCATTCCAG
	TCTAAAGCGTTCCCTCCTCACTGTGGTCCTAGTCAATGCG
Probe-3	TGGTAGGTAGCGTTTCCAGT
	ATTTCCTGAATTCCTGGAATAAAACAAAACAAAAAAAAG
Probe-4	CCCCCAAACAAAACTCTATCA
	GGAAGTCGTGGTTTTCTGTAAAAAAAGGTTTTACACATC
Probe-5	AGAAATCGGACTTCTGGGGCA
	AGCTGCAGTGTTTTCCTCTTCATGCCAAAGAGATTAATAT
Probe-6	TTACGGTGAAGACTTTTCCA
	ATCGAAAGGGAAAAAATAAAAGAAATTGCAGTTTATATT
Probe-7	TTTGTTATCCCATGAAAAATT
	GGTACAAAATTTGGAAAATGGACCTGGCGTTGAAATCTT
Probe-8	TACTCCTCGTTTAAAAAGACC
	GTTCCGGAGAGGGTTCGGTCTGCGGAGAAGCGCGCGAGA
Probe-9	GACAAGCATCCTCAAGGTTCT
	GCCACCCTCAGGCTCAAGTCCCAGGAGGCCTCCTCCGCT
Probe-10	GACTTGGGTGAACAGCTCTGA
	ATGTTCAAGCTCCGCGTCACTCATCGGTACCAAGTTGATG
Probe-11	ATCTCTGCCTAGGGTTCTTC
	GCCTCTTGCAGTTAACACTCAGCCTCTTAACTGGGAGTGG
Probe-12	CCCTGGAGCAAGTCGCTGGA
	GACTGGGTCTGTATCCAAACCCGGTTCGGTTCCTGGAGC
Probe-13	CAACGGCGTAGTTAACACTCA
	TTACTAAAGCGTCATTTAACAATTTTTCATATTATAGAAA
Probe-14	CAGCTTTTCATGGATGGTGT
	TTGTTTGGAAAGATGAAAAATTTATTAGAAAAGATCTTT
Probe-15	AAATGGCTT

a: F, Forward; b: R, Reverse.
